# ﻿Battle of the bands: systematics and phylogeny of the white *Goniobranchus* nudibranchs with marginal bands (Nudibranchia, Chromodorididae)

**DOI:** 10.3897/zookeys.1083.72939

**Published:** 2022-01-25

**Authors:** Giun Yee Soong, Lynn J. Bonomo, James D. Reimer, Terrence M. Gosliner

**Affiliations:** 1 Molecular Invertebrate Systematics and Ecology Laboratory, Graduate School of Engineering and Science, University of the Ryukyus, Senbaru 1, Nishihara, Okinawa 903–0213, Japan; 2 Department of Invertebrate Zoology & Geology, California Academy of Sciences, San Francisco, California 94118, USA; 3 Tropical Biosphere Research Center, University of the Ryukyus, Senbaru 1, Nishihara, Okinawa 903–0213, Japan

**Keywords:** Biodiversity, coral reefs, mtDNA, species delimitation, taxonomy

## Abstract

Species identities of *Goniobranchus* nudibranchs with white bodies and various marginal bands have long been problematic. In this study, specimens of these *Goniobranchus* nudibranchs from the Philippines, Peninsular Malaysia, Japan, Papua New Guinea, and Madagascar were analyzed and molecular data were obtained in order to re-examine the relationships between species within this “white *Goniobranchus* with marginal bands” group. The analyses clearly recovered six species groups corresponding to the described species *Goniobranchusalbonares*, *G.preciosus*, *G.rubrocornutus*, *G.sinensis*, and *G.verrieri* as well as one new species, *G.fabulus* Soong & Gosliner, **sp. nov.** Notably, *G.preciosus*, *G.sinensis*, *G.rubrocornutus*, *G.verrieri*, and *G.fabulus* Soong & Gosliner, **sp. nov.** exhibit color variation and polymorphism, suggesting that some aspects of color patterns (e.g., presence or absence of dorsal spots) may not always be useful in the identification of species in the “white *Goniobranchus* with marginal bands” group, whereas other features such as gill and rhinophore colors and the arrangement and colors of the mantle marginal bands are more diagnostic for each species.

## ﻿Introduction

Research focusing on the diversity within Nudibranchia through molecular work has increased in recent years (e.g., Epstein et al. 2019; Korshunova et al. 2020), and a better understanding of the phylogenetic relationships within the clade has been achieved via molecular phylogenetic analyses. These studies have also revealed new species, many of which have been cryptic or pseudocryptic species or members of species complexes (e.g., Layton et al. 2018; Matsuda and Gosliner 2018a; Epstein et al. 2019; Sørensen et al. 2020). The genus *Goniobranchus* was previously synonymized with the genus *Chromodoris*, but molecular analyses by Johnson and Gosliner (2012) revealed that *Chromodoris* was non-monophyletic. This resulted in the generic reinstatement of *Goniobranchus* for one of the distinct clades of *Chromodoris* (Johnson and Gosliner 2012), but morphological differences are not clear. *Goniobranchus* currently contains 57 described species (MolluscaBase 2021), and members of this genus can be identified for laying raised egg masses (i.e., one edge of the egg mass is attached to the substrate, while the other stands up in the water column).

Within *Goniobranchus* there are several species complexes, each containing similar species grouped together based on their external coloration and patterns, and many times involving cryptic or pseudocryptic species (Johnson and Gosliner 2012; Soong et al. 2020). One such group is the red-reticulate species complex with three described species and several synonymies that were summarized by Rudman (1973). A recent molecular phylogenetic examination revealed the presence of five potentially undescribed species within this species complex that are cryptic with the described species (Soong et al. 2020).

Another likely pseudocryptic *Goniobranchus* species complex contains species with white bodies and variously colored marginal bands. This group has not been thoroughly examined through molecular sequencing. Rudman (1985) provided the most recent taxonomic assessment on this species complex and placed *Goniobranchuspreciosus* (Kelaart, 1858), *G.verrieri* (Crosse, 1875), *G.trimarginatus* (Winckworth, 1946), *G.sinensis* (Rudman, 1985), *G.rubrocornutus* (Rudman, 1985), and *G.galactos* (Rudman & Johnson, 1985) within the group. Gosliner et al. (2015) subsequently included *G.albonares* (Rudman 1990) in the complex due to a similarity in color patterns. Since all these species share similar colors and patterns on their bodies, and as the group has a large geographic range from the Indian Ocean to the Western Pacific Ocean (Debelius 1996; Debelius and Kuiter 2007; Coleman 2008; Gosliner et al. 2008, 2015, 2018), it has been postulated that undescribed, cryptic species may exist within this group (Rudman 1985; Gosliner et al. 2008, 2015, 2018). However, previous taxonomic studies on this group of *Goniobranchus* focused only on morphological analyses and most of the previous molecular sequences were by Johnson and Gosliner (2012) who included only a few representatives of this particular group, namely *G.sinensis*, *G.preciosus*, *G.verrieri*, and *G.daphne*; they recovered a monophyletic group of species with white bodies with variously colored marginal bands in their study. Here, we incorporate molecular data to re-examine the phylogenetic relationships between several putative *Goniobranchus* species with white bodies and variously colored marginal bands and, as a result of our phylogenetic and morphological analyses, we formally describe one novel species. Specimens of two species that Rudman (1985) included in his study (*G.galactos* and *G.trimarginatus*) were not included in the present study, as no material appropriately fixed for molecular sequencing was available.

## ﻿Materials and methods

### ﻿Taxon sampling

A total of 35 *Goniobranchus* specimens with white mantles and various marginal bands was examined in this study (Table 1). The specimens were either deposited in the California Academy of Sciences Invertebrate Zoology collection or newly collected from Kagoshima and Okinawa in southern Japan by SCUBA diving (Table 1). Additionally, sequences from specimens of *Glossodoris* species (*G.bonwanga*, *G.andersonae*, *G.buko*, *G.cincta*, *G.pallida*, *G.acosti*, and *G.hikuerensis*) were used as the outgroup in our analyses, based on the most recently published family Chromodorididae phylogeny (Johnson and Gosliner 2012). Specimens were photographed in situ before collection and fixation, in either 95% or 99.5% ethanol for DNA molecular work or 10% formalin for morphological work. All specimens were preliminarily identified based on their external morphologies and subsequent identifications were made by the senior author.

**Table 1.** List of specimens used in this study. ﻿Asterisk indicates sequence acquired from GenBank. Institution and voucher codes: CASIZ (California Academy of Sciences Invertebrate Zoology), WAM (Western Australian Museum), SAM (South Australian Museum), UQ (University of Queensland), MISE (Molecular Invertebrate Systematics and Ecology), Okinawa, Japan.

Species name	Morpho–type	Voucher number	Location	Depth (m)	GenBank accession numbers	
					COI	16S
**Outgroups**						
*Glossodorisacosti*	–	CASIZ 175327*	Bohol Island, Philippines	1–5	KT600696	KT595626
*Glossodorisandersonae*	–	CASIZ 192288*	Abulad Islands, Saudi Arabia	7	KT600694	KT595623
*Glossodorisbonwanga*	–	CASIZ 194018*	South Madagascar, Madagascar	3–8	KT600695	KT595647
*Glossodorisbuko*	–	CASIZ 177408*	Batangas Province, Philippines	21	KT600711	KT595638
*Glossodoriscincta*	–	CASIZ 177257*	Batangas Province, Philippines	14	KT600700	KT595627
*Glossodorishikuerensis*	–	CASIZ 116935*	Kwajalein Atoll, Marshall Islands	16	KT600704	KT595632
**Ingroups**						
*Goniobranchusalbonares*	–	CASIZ 191440	Madang Province, Papua New Guinea	–	OL685221	OL684806
*Goniobranchusalbonares*	–	CASIZ 228939	Batangas Province, Philippines	5	OL685222	OL684786
*Goniobranchusalbonares*	–	CASIZ 194037	South Madagascar, Madagascar	22	OL685223	OL684810
*Goniobranchusalbonares*	–	N/A*	New South Wales, Australia	–	KJ001299	KJ018909
*Goniobranchusalbopunctatus*	–	CASIZ 121268*	Western Australia, Australia	30	JQ727827	JQ727700
*Goniobranchusalbopustulosus*	–	CASIZ 142953*	Maui, Hawaiʻi	7	JQ727828	JQ727701
*Goniobranchusaureopurpureus*	–	N/A*	–	–	EU512128	EU512055
*Goniobranchuscoi*	–	CASIZ 158683*	Batangas Province, Philippines	20	EU982734	EU982785
*Goniobranchuscoi*	–	N/A*	–	–	EU512144	EU512061
*Goniobranchuscollingwoodi*	–	CASIZ 139597*	Bali, Indonesia	24	JQ727834	JQ727710.1
Goniobranchuscf.collingwoodi	–	CASIZ 159382*	Queensland, Australia	–	JQ727835	JQ727711
*Goniobranchusdaphne*	–	UQ 802*	Tasmania, Australia	5	MH018004	MH017991
*Goniobranchusdaphne*	–	N/A*	Queensland, Australia	–	KJ001297	KJ018921
*Goniobranchusdecorus*	–	N/A*	–	–	EU512146	EU512068
*Goniobranchusdecorus*	–	CASIZ 157025*	Batangas Province, Philippines	8	EU982735	EU982786
*Goniobranchusepicurius*	–	SAM D19285*	Tasmania, Australia	–	EF535114	AY458804
*Goniobranchusfabulus*	A	CASIZ 177517	Batangas Province, Philippines	–	OL685216	OL684785
*Goniobranchusfabulus*	A	CASIZ 201949	Batangas Province, Philippines	–	OL685224	OL684787
*Goniobranchusfabulus*	A	CASIZ 177685	Batangas Province, Philippines	15	OL685217	OL684807
*Goniobranchusfabulus*	B	CASIZ 191271	Madang Province, Papua New Guinea	–	OL685220	OL684804
*Goniobranchusfabulus*	B	CASIZ 191118	Madang Province, Papua New Guinea	3	OL685219	OL684805
*Goniobranchusfidelis*	–	CASIZ 175556*	Iles Radama, Madagascar	30	JQ727839	JQ727714
*Goniobranchusfidelis*	–	CASIZ 175426*	Batangas Province, Philippines	–	JQ727838	JQ727715
*Goniobranchusgeminus*	–	CASIZ 173434*	Iles Radama, Madagascar	13–16	JQ727840	JQ727716
*Goniobranchusgeometricus*	–	CASIZ 144023*	Queensland, Australia	11	JQ727841	JQ727718
*Goniobranchusgeometricus*	–	CASIZ 177549*	Batangas Province, Philippines	22.7	JQ727842	JQ727717
*Goniobranchusgeometricus*	–	MO6*	North Sulawesi, Indonesia	> 6	MK348906	MK322449
*Goniobranchusgeometricus*	–	Goge 16S1*	North Sulawesi, Indonesia	6–19	MN339442	MN104715
*Goniobranchusgeometricus*	–	Goge 16S2*	North Sulawesi, Indonesia	6–19	MN339443	MN104716
*Goniobranchusgeometricus*	–	Goge 16S3*	North Sulawesi, Indonesia	6–19	MN339444	MN104717
*Goniobranchusheatherae*	–	CASIZ 175546*	Cape Peninsula, South Africa	–	JQ727844	JQ727720
*Goniobranchushintuanensis*	–	CASIZ 158346*	Batangas Province, Philippines	10	JQ727845	JQ727721
*Goniobranchushunterae*	–	UQ 915*	Tasmania, Australia	–	MH018008	MH017995
*Goniobranchushunterae*	–	UQ 824*	Tasmania, Australia	–	MH018006	MH017993
*Goniobranchusleopardus*	–	CASIZ 159384*	Queensland, Australia	16	JQ727847	JQ727726
*Goniobranchusleopardus*	–	SAM D 19288*	Queensland, Australia	–	EF535116	AY458808
*Goniobranchusloringi*	–	WAM S111031*	New South Wales, Australia	–	MH018013	MH018000
*Goniobranchuspreciosus*	A	CASIZ 208420	Oriental Mindoro Province, Philippines	4–22	OL685227	OL684811
*Goniobranchuspreciosus*	A	CASIZ 208415	Oriental Mindoro Province, Philippines	–	OL685226	OL684794
*Goniobranchuspreciosus*	B	CASIZ 208574	Oriental Mindoro Province, Philippines	6–16	OL685230	OL684813
*Goniobranchuspreciosus*	C	CASIZ 176752	Pulau Tioman, Peninsular Malaysia	13	OL685213	OL684815
*Goniobranchuspreciosus*	D	CASIZ 176761	Pulau Tioman, Peninsular Malaysia	17	OL685215	OL684814
Goniobranchuscf.roboi	–	CASIZ 121275*	Rottnest Island, Australia	30	JQ727854	JQ727734
*Goniobranchusrubrocornutus*	A	CASIZ 203047	Batangas Province, Philippines	–	OL685225	OL684782
*Goniobranchusrubrocornutus*	B	CASIZ 208563	Oriental Mindoro Province, Philippines	18	OL685229	OL684783
*Goniobranchusrufomaculatus*	–	N/A*	–	–	EU512131	EU512057
*Goniobranchussinensis*	A	CASIZ 176759	Pulau Tioman, Peninsular Malaysia	13	OL685214	OL684793
*Goniobranchussinensis*	A	CASIZ 175727	Pulau Tioman, Peninsular Malaysia	13	OL685212	OL684792
*Goniobranchussinensis*	A	CASIZ 189457	Pulau Tioman, Peninsular Malaysia	–	OL685218	OL684809
*Goniobranchussinensis*	B	MISE–KS008–19	Okinawa, Japan	8	OL685232	OL684795
*Goniobranchussinensis*	B	MISE–KS009–19	Okinawa, Japan	8	OL685233	OL684796
*Goniobranchussinensis*	B	MISE–KS010–19	Okinawa, Japan	8	OL685234	OL684797
*Goniobranchussinensis*	B	MISE–KS018–19	Okinawa, Japan	6	OL685235	OL684798
*Goniobranchussinensis*	B	MISE–KS020–18	Okinawa, Japan	9	OL685236	OL684799
*Goniobranchussinensis*	B	MISE–KS021–18	Okinawa, Japan	10	OL685237	OL684800
*Goniobranchussinensis*	B	MISE–KS022–18	Okinawa, Japan	10	OL685238	OL684801
*Goniobranchussinensis*	B	MISE–KS023–18	Okinawa, Japan	9	OL685239	OL684802
*Goniobranchussinensis*	B	MISE–KS024–18	Okinawa, Japan	–	OL685240	OL684803
*Goniobranchussinensis*	B	MISE–KS024–19	Okinawa, Japan	5	OL685241	OL684784
*Goniobranchussinensis*	B	MISE–KS055–19	Okinawa, Japan	–	OL685245	OL684790
*Goniobranchussinensis*	B	MISE–KS056–19	Okinawa, Japan	12	OL685246	OL684791
*Goniobranchussinensis*	C	MISE–KS037–19	Kagoshima, Japan	–	OL685242	OL684788
*Goniobranchussinensis*	C	MISE–KS039–19	Kagoshima, Japan	8	OL685243	OL684808
*Goniobranchussinensis*	C	MISE–KS047–19	Kagoshima, Japan	–	OL685244	OL684789
*Goniobranchussplendidus*	–	CASIZ 146039*	Queensland, Australia	21	EU982738	EU982789
*Goniobranchussplendidus*	–	UQ 1102*	Queensland, Australia	–	MH018011	MH017998
*Goniobranchussplendidus*	–	SAM D19292*	Queensland, Australia	–	EF535115	AY458815
*Goniobranchustasmaniensis*	–	UQ 892*	Tasmania, Australia	–	MH018007	MH017994
*Goniobranchustasmaniensis*	–	SAM D19295*	Tasmania, Australia	–	EF535113	AY458817
Goniobranchusaff.tinctorius	–	WAM S71088*	Queensland, Australia	–	MH018010	MH017997
Goniobranchusaff.tinctorius	–	CASIZ 156921*	Batangas Province, Philippines	–	JQ727853	JQ727733
Goniobranchusaff.tinctorius	–	N/A*	Queensland, Australia	–	KJ001315	KJ018910
Goniobranchusaff.tinctorius	–	Gore 16Sa1*	North Sulawesi, Indonesia	6–9	MN339446	MN104719
Goniobranchusaff.tinctorius	–	Gore 16Sa2*	North Sulawesi, Indonesia	6–9	MN339447	MN104720
*Goniobranchusverrieri*	Unknown	CASIZ 158796*	Batangas Province, Philippines	–	JQ727858	JQ727740
*Goniobranchusverrieri*	A	CASIZ 203059	Batangas Province, Philippines	–	OL685231	OL684816
*Goniobranchusverrieri*	B	CASIZ 208442	Batangas Province, Philippines	3–30	OL685228	OL684812
*Goniobranchusvibratus*	–	CASIZ 175564*	Hawaiʻi, USA	–	JQ727859	JQ727741
*Goniobranchuswoodwardae*	–	N/A*	–	–	EU512127	EU512103

### ﻿DNA extraction, amplification, sequencing

DNA was extracted from the *Goniobranchus* specimen tissues using a Qiagen DNeasy Blood and Tissue Kit (Qiagen, Tokyo, Japan) either at the Molecular Invertebrate Systematics and Ecology (MISE) Laboratory (Okinawa, Japan) or at the California Academy of Sciences Center for Comparative Genomics (CCG; San Francisco, CA, USA). Polymerase chain reaction (PCR) amplifications for specimens deposited in the California Academy of Sciences Invertebrate Zoology were done following a protocol used by Bonomo and Gosliner (2020). PCR amplifications for the remaining specimens were performed at the Molecular Invertebrate Systematics and Ecology Laboratory using 20 μL reaction volume, consisting of 7 μL H2O, 10 μL Hot Start Taq Plus Master Mix Kit (Qiagen, Tokyo, Japan), 1 μL of each primer and 1 μL of genomic DNA. Two mitochondrial genes, cytochrome c oxidase subunit I (COI) and 16S ribosomal RNA (16S rDNA), were amplified. The universal primers used for COI were LCO1490 (5'-GGTCAACAAATCATAAAGATATTGG-3') and HCO2198 (5'-TAAACTTCAGGGTGACCAAAAAATCA-3') from Folmer et al. (1994). The universal primers used for 16S were 16Sar-L (5'-CGCCTGTTTATCAAAAACAT-3') by Palumbi et al. (1991) and 16SR (5'-CCGGTTTGAACTCAGATCATGT-3') from Palumbi (1996). The targeted fragment length for COI was 658 base pairs and for 16S was 560 base pairs. The COI amplification started with an initial denaturation of 94 °C for 3 mins; 39 cycles of denaturation at 94 °C for 30 s, annealing at 46 °C for 30 s, an extension at 72 °C for 60s, and then a final extension at 72 °C for 5 mins. The 16S amplification started with an initial denaturation of 94 °C for 3 mins; 39 cycles of denaturation at 94 °C for 30 s, annealing at 52 °C for 30 s, an extension at 72 °C for 60 s; and a final extension at 72 °C for 5 mins and 25 °C for 60 s. The amplification parameters were based on Johnson and Gosliner (2012). All PCR products that were successfully amplified were cleaned and purified using Exonuclease I – Shrimp Alkaline Phosphatase (ExoSAP) and they were either sequenced at the CCG or sent to FASMAC (Kanagawa, Japan) for sequencing in both directions.

### ﻿Phylogenetic analyses

The sequences obtained were assembled, trimmed, and edited in Geneious v. 10.2.3 (Kearse et al. 2012). Publicly available COI and 16S GenBank sequences for *Goniobranchus* species were included in our dataset for analyses (Table 1). In total, 89 taxa were analyzed, and the alignment of sequences was done using MAFFT v. 7.450 (Katoh and Standley 2013) within Geneious. The alignments of each gene were trimmed to 569 and 476 base pairs, respectively, for COI and 16S. Thus, the concatenated dataset included 1,045 base pairs in total for 89 taxa.

Maximum likelihood (ML) and Bayesian inference (BI) were used to construct the phylogenetic trees among species for both markers as well as the concatenated data (COI+16S). The RAxML Next Generation (RAxML-NG) v. 1.0.2 (Kozlov et al. 2019) was used to run the ML analyses on our COI and 16S dataset using TIM1+I+G and TVM+I+G model respectively with 1000 bootstrap replications. MrBayes v. 3.2.6 (Huelsenbeck and Ronquist 2001) was used to perform the BI analyses on the same dataset using the HKY+I+G and GTR+G model for COI and 16S partitions, respectively. The best evolutionary models were determined using TOPALi (Milne et al. 2009). The Bayesian Markov chain Monte Carlo (MCMC) was run for 5 × 10^6^ generations where chains were sampled every 200 generations. A standard 25% burn-in length was removed from the dataset, at which point the Average Standard Deviation of Split Frequency (ASDSF) was < 0.01.

### ﻿Species delimitation

Automatic Barcode Gap Discovery (ABGD) (Puillandre et al. 2012) uses genetic pairwise differences to determine species-level clusters based on “barcode gaps”. The ABGD analyses of our COI and 16S dataset were performed online (https://bioinfo.mnhn.fr/abi/public/abgd/abgdweb.html) and the following parameters were applied: P_min_ = 0.001, P_max_ = 0.1, Steps = 10, *X* = 1, and Nb bins = 20 using the Jukes-Cantor (JC69) model. The uncorrected pairwise *p*-distances for COI were also calculated in MEGA v. 6.06 (Tamura et al. 2013).

### ﻿Morphology

Based on the ABGD analyses, selected representative specimens from each delimited species-level clade were morphologically examined. The specimens’ rhinophores and gill structures were examined, as well as their reproductive systems and buccal masses. The morphologies of all specimens were also compared with all known species descriptions of *Goniobranchus* species with white mantles and various marginal bands.

The reproductive system and buccal mass for each specimen were dissected using a Nikon SMZ-U dissecting scope. The buccal mass was extracted and placed into a concentrated 10% sodium hydroxide solution for 24 hours. Connective tissues on the radula and jaw were carefully removed with the aid of a dissecting microscope. The jaw and radula were then rinsed with distilled water and mounted on a glass slide to dry. To view the radula and jaw under the scanning electron microscope, the radula and jaw were placed on a stub that was placed in a sputter coater (Cressington 108 Auto vacuum sputter coater) to cover the specimen with a thin layer of gold/palladium. For observation, we used a scanning electron microscope (Hitachi SU35), and the number and shape of the teeth were observed from the images.

The reproductive systems that were extracted from the specimens were hand drawn under a dissecting microscope (Nikon SMZ-U) with a camera lucida attached. The shape and size of the organs in the reproductive system were noted and illustrated.

## ﻿Results

### ﻿Phylogenetic and species delimitation analyses

A total of 35 new sequences was obtained for both COI and 16S genes (Table 1). The alignments of each gene were trimmed to 569 and 476 base pairs, respectively. Combined with sequences from GenBank, the concatenated dataset included 1,045 base pairs in total for 89 taxa. The ABGD analysis of the COI alignment recovered six species-level clades and the prior maximal distances, *P*, were stable from 0.0028 to 0.0046. The 16S dataset also recovered the same six species-level clades and the prior maximal distances, *P*, were stable from 0.0046 to 0.0077. The groups within the complex recovered were *G.albonares* (*n* = 4), *G.daphne* (*n* = 2), *G.verrieri* (*n* = 3), *G.preciosus* (*n* = 5), *G.rubrocornutus* (*n* = 2), *G.sinensis* (*n* = 18), and *G.fabulus* sp. nov. (*n* = 5), with interspecific *p*-COI distances ranging from 2.5–18.6% (Table 2).

**Table 2.** Interspecific and intraspecific range of distances among and within clades in percentages (%).

	*Goniobranchusalbonares*	*Goniobranchuspreciosus*	*Goniobranchusrubrocornutus*	*Goniobranchussinensis*	*Goniobranchusverrieri*	*Goniobranchusfabulus* sp. nov.	*Goniobranchusdaphne*
*Goniobranchusalbonares*	1.1–5.2	–	–	–	–	–	–
*Goniobranchuspreciosus*	15.5–18.6	0.4–2.7	–	–	–	–	–
*Goniobranchusrubrocornutus*	14.8–16.1	9.9–10.8	0.0	–	–	–	–
*Goniobranchussinensis*	14.3–16.6	7.1–9.8	9.6–11.2	0.0–1.4	–	–	–
*Goniobranchusverrieri*	16.0–18.2	10.8–12.6	10.7–11.8	10.0–12.1	1.3–3.7	–	–
*Goniobranchusfabulus* sp. nov.	14.0–18.2	6.8–9.2	7.8–9.3	6.3–8.6	10.1–12.0	0.2–3.4	–
*Goniobranchusdaphne*	15.3–17.5	7.4–7.9	8.9–9.0	6.7–8.5	10.8–11.4	2.5–4.5	0.5

**Figure 1.**
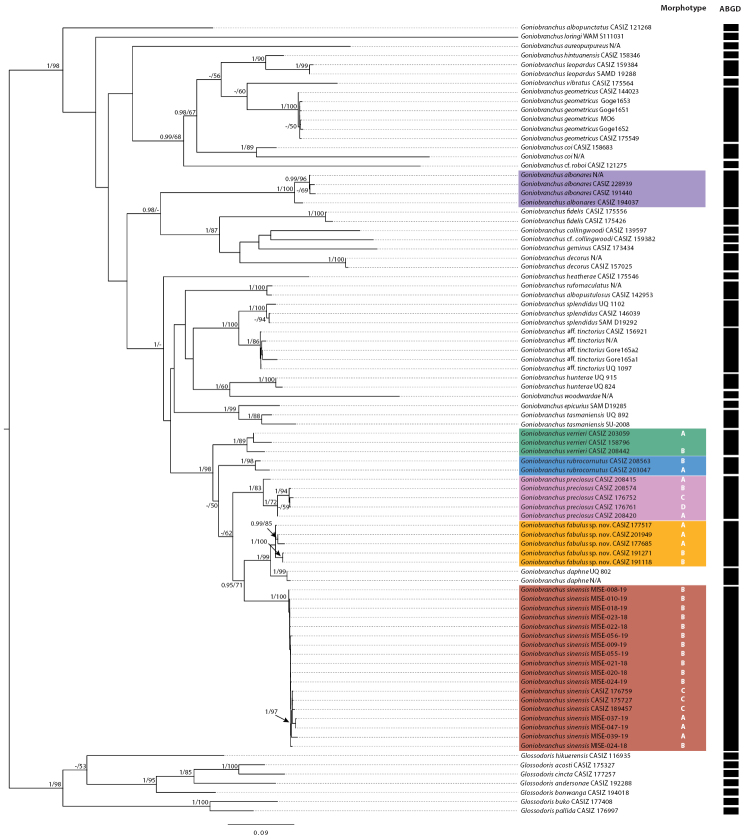
Molecular phylogeny based on the combined dataset (COI+16S rDNA) inferred by maximum likelihood (ML) and Bayesian inference (BI). Numbers on nodes represent Bayesian posterior probabilities (> 0.95) / ML bootstrap values (only > 50% values are shown). Black bars indicate the clade groupings of ABGD analysis on the COI + 16S dataset.

In the concatenated COI+16S tree (Fig. 1), two monophyletic clades containing members of the white *Goniobranchus* with marginal bands group were recovered. The first clade, including specimens identified as *Goniobranchusalbonares*, *G.collingwoodi*, *G.decorus*, *G.fidelis*, and *G.geminus*, was well-supported (0.98/-%, Bayes and ML, respectively) and its sister group, a clade which included specimens identified as *G.verrieri*, *G.rubrocornutus*, *G.preciosus*, *G.fabulus* sp. nov., *G.daphne*, and *G.sinensis* plus *G.albopustulosus*, *G epicurius*, *G.heatherae*, *G.hunterae*, *G.rufomaculatus*, *G.splendidus*, *G.tasmaniensis*, G.aff.tinctorius, as well as *G.woodwardae* had moderate support (1/48%). The “white body and variously colored marginal bands” species formed a monophyletic group with the exception of *G.albonares*, which was closely related to *G.fidelis* that is not a “white body and variously colored marginal bands” species. However, *G.verrieri*, *G.rubrocornutus*, *G.preciosus*, *G.fabulus* sp. nov., *G.daphne*, and *G.sinensis*, which are all part of the group in question, formed a well-supported monophyletic clade (1/98%). The clade of *G.albonares* specimens was strongly supported (1/100%) and was sister to a clade of *G.collingwoodi*, *G.decorus*, *G.fidelis*, and *G.geminus*. The second main clade contained members of the white *Goniobranchus* with marginal bands group and also contained *G.albopustulosus*, *G epicurius*, *G.heatherae*, *G.hunterae*, *G.rufomaculatus*, *G.splendidus*, *G.tasmaniensis*, and G.aff.tinctorius as well as *G.woodwardae* with moderate support (1/48%). However, none of these other members of this second clade have a series of marginal bands. Within the well-supported monophyletic white *Goniobranchus* with marginal bands group subclade (1/98%), *G.verrieri* (1/89%) was sister to *G.rubrocornutus*, *G.preciosus*, *G.fabulus* sp. nov., *G.daphne*, and *G.sinensis*. A well-supported *G.preciosus* (1/83%) was sister to *G.fabulus* sp. nov., *G.daphne*, and *G.sinensis*. A well-supported subclade containing *G.fabulus* sp. nov. and *G.daphne* (1/99%) formed a sister clade to a well-supported *G.sinensis* subclade (1/100%). Additionally, there were no color morphs of any species observed that mimicked the coloration patterns of another species. This is the opposite of what has been seen in other groups of chromodorid nudibranchs, for example in *Chromodoris* (Layton et al. 2018, 2020). The confusion between the species studied here is due to a misperception regarding the morphological attributes of each species and concerning what color patterns hold constant across a species.

### ﻿Morphological analyses

**Figure 2.**
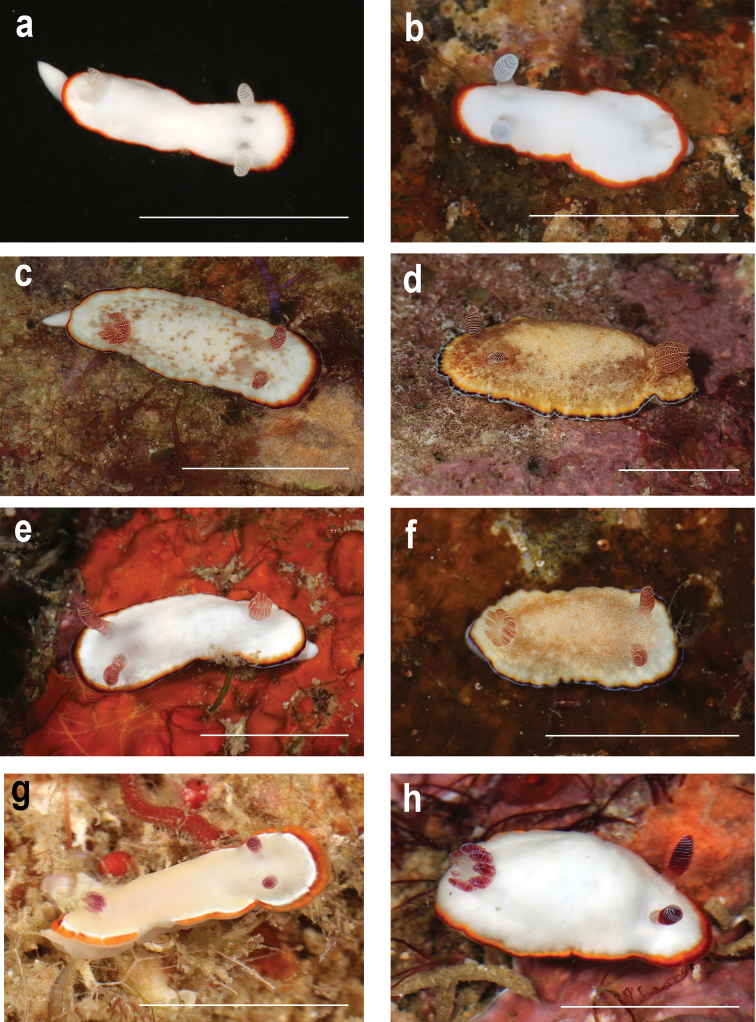
**a, b***Goniobranchusalbonares***a**CASIZ 191440, Papua New Guinea **b**CASIZ 228939, Philippines **c–f***Goniobranchuspreciosus***c**CASIZ 208415, Morphotype A, Philippines **d**CASIZ 208574, Morphotype B, Philippines **e**CASIZ 176752, Morphotype C, Peninsular Malaysia **f**CASIZ 176761, Morphotype D, Peninsular Malaysia **g, h***Goniobranchusrubrocornutus***g**CASIZ 203047, Morphotype A, Philippines **h**CASIZ 208563, Morphotype B, Philippines. Photographs TMG. Scale bars: 1 cm.

**Figure 3.**
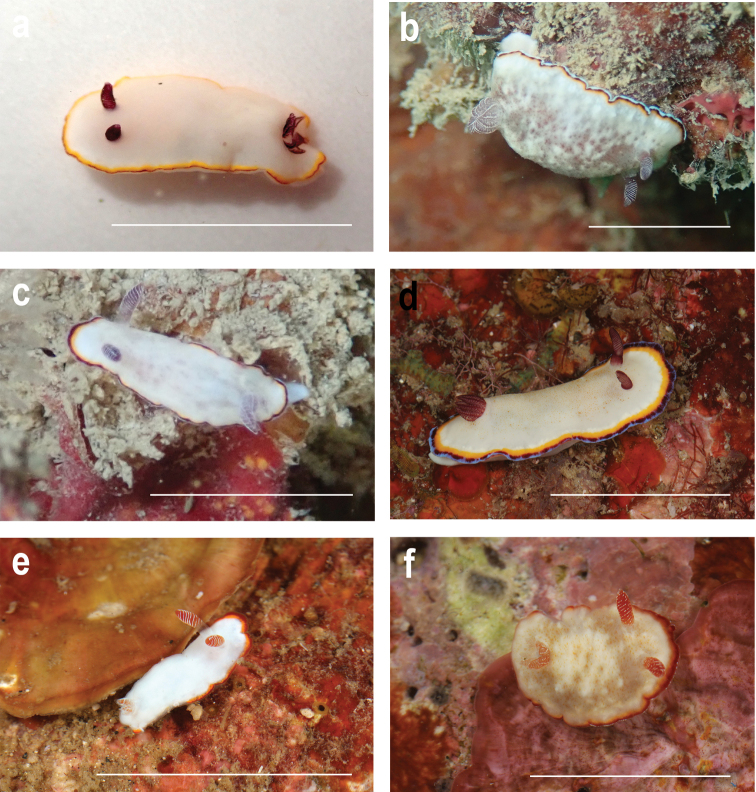
**a–d***Goniobranchussinensis***a**CASIZ 176759, morphotype A, Peninsular Malaysia **b**MISE-018-19, morphotype B, Okinawa, Japan **c**MISE-55-19, morphotype B, Okinawa, Japan **d**MISE-039-19, morphotype C, Kagoshima, Japan **e, f***Goniobranchusverrieri***e**CASIZ 203059, morphotype A, Philippines **f**CASIZ 208442, morphotype B, Philippines. Photographs **a, e, f** TMG; **b–d** GYS. Scale bars: 1 cm.

**Figure 4.**
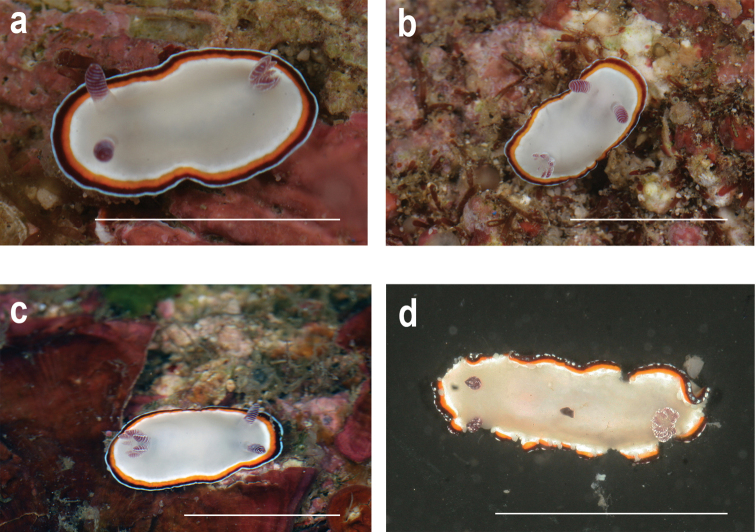
*Goniobranchusfabulus* sp. nov. **a**CASIZ 177517, morphotype A, Philippines **b**CASIZ 177685, morphotype A, Philippines **c**CASIZ 201949, morphotype A, Philippines **d**CASIZ 191118, morphotype B, Papua New Guinea. Photographs TMG. Scale bars: 1 cm.

The species recovered from the phylogenetic and ABGD analyses are shown in Figure 1, whereas morphotypes are shown in Figures 2–4. Most of the species in this study demonstrated high levels of morphological variation. Each of *G.rubrocornutus*, *G.preciosus*, *G.fabulus* sp. nov., *G.verrieri*, and *G.sinensis* showed at least two distinct morphotypes that had no significant genetic differences between morphotypes (Table 2).

In terms of jaw and radular morphology, all specimens had bifid rodlets and one distinctive rachidian tooth except for *G.rubrocornutus*, which is shown to have a very thin rachidian tooth that can easily pass unnoticed (Fig. 10d). In some species, while external morphology was variable, aspects of the external color pattern, radular morphology, and their reproductive anatomy exhibited clear and distinct differences, which are detailed in the following systematics section.

## ﻿Systematics

### Family Chromodorididae Bergh, 1891

#### ﻿Genus Goniobranchus

Taxon classificationAnimaliaNudibranchiaChromodorididae

Pease, 1866

D228344C-FFA1-5742-8068-A778BB6144F1

##### Type species.

*Dorisvibrata* Pease, 1860 = *Goniobranchusvibratus* (Pease, 1860) by monotypy. Type locality: Hawaiʻi.

#### ﻿ Goniobranchus albonares

Taxon classificationAnimaliaNudibranchiaChromodorididae

(Rudman, 1990)

62448D73-B803-53FE-8229-D761BE07A3CA

**Figure 5.**
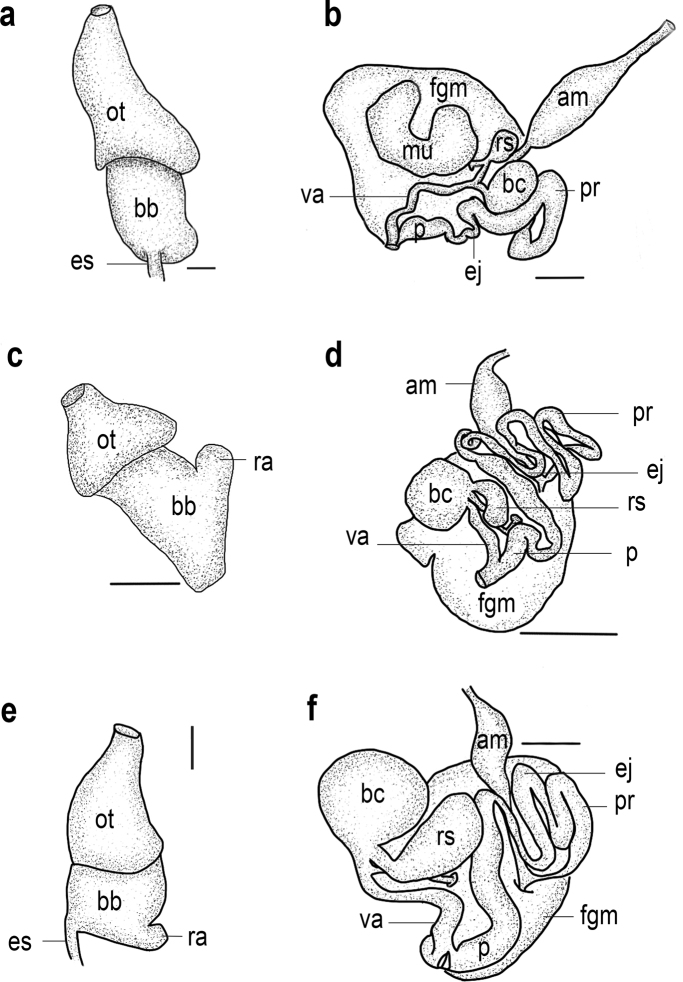
**a** buccal mass of *Goniobranchusalbonares*, CASIZ 228939 **b** reproductive system of *Goniobranchusalbonares*, CASIZ 228939 **c** buccal mass of *Goniobranchuspreciosus*, CASIZ 208574 **d** reproductive system of *Goniobranchuspreciosus*, CASIZ 208574 **e** buccal mass of *Goniobranchusrubrocornutus*, CASIZ 203047 **f** reproductive system of *Goniobranchusrubrocornutus*, CASIZ 203047. Abbreviations: am, ampulla; bb, buccal bulb; bc, bursa copulatrix; ej, ejaculatory duct; es, esophagus; fgm, female gland mass; ot, oral tube; p, penis; pr, prostate; ra, radular sac; rs, receptaculum seminis; va, vagina; mu, mucous gland. Scale bars: 0.1 mm (**a, b, e, f**); 1 mm (**c, d**).

Figures 2a, b , 5a, b , 7a–f

Chromodoris albonares Rudman, 1990: 100, 307–309, figs 26E, 35, 36; Gosliner et al. 2008: 220, second photograph from the top.
Goniobranchus albonares : Gosliner et al. 2015: 223, lower left photograph; Gosliner et al. 2018: 153, lower left photograph.

##### Type locality.

New South Wales, Australia.

##### Type material.

AM C156989, one specimen, west side of Northwest Solitary Island, 30.017°S, 156.267°E, Coffs Harbour, New South Wales, Australia, 6 m depth, 4 December 1988, J. & J. England, P. Edwards. Not examined in this study due to the original descriptions in Rudman (1990) being comprehensive.

##### Geographical distribution.

Widely distributed around the tropical and subtropical Indo-Pacific Ocean (Debelius and Kuiter 2007; Gosliner 2008, 2015, 2018), Mozambique (Tibiriçá et al. 2017; Strömvoll and Jones 2019), Indonesia (Debelius and Kuiter 2007), Japan (Nakano 2018; Ono and Katou 2020), Taiwan (Jie et al. 2009), Australia (Rudman 1990), Madagascar, Philippines, Papua New Guinea (present study), New Caledonia (Hervé 2010), and Gulf of Oman (Fatemi and Attaran-Fariman 2015).

##### Material examined.

CASIZ 228939, one specimen (2 mm preserved), subsampled for molecular data and dissected, Murals dive site, 13.688°N, 120.866°E, Maricaban Strait, Mabini (Calumpan Peninsula), Batangas Province, Luzon, Philippines, 9–22 m depth, 29 November 2018, T.M. Gosliner, 2018 Verde Island Passage Expedition. CASIZ 191440, one specimen (3 mm preserved), subsampled for molecular data, Madang Province, GPS not available, Papua New Guinea, depth not available, 26 November 2012, V. Knutson, Papua New Guinea Biodiversity Expedition 2012. CASIZ 194037, one specimen (2 mm preserved), subsampled for molecular data, Pointe Evatra, rocky bottom with areas of sand, 24.983°S, 47.083°E, South Madagascar, Madagascar, 22 m depth, 30 April 2010, *Atimo Vatae* South Madagascar Expedition.

##### Description.

***External morphology*.** Living animals 5–7 mm in length. Body opaque white, oval and elongated, with the outermost portion of the mantle edge having an orange band that gradually blends into a yellow submarginal band. Gill and rhinophores are translucent white with opaque white edges on the lamellae. Six or seven unipinnate gill branches are moderately spreading when fully extended. Rhinophores are relatively large, ~ 2× as long as the gill branches. Ten or eleven lamellae per rhinophore.

***Buccal mass and radula*.** The muscular portion of the buccal mass ~ 2/3 the size of the oral tube length (Fig. 5a). The chitinous labial cuticle found at the anterior end of the muscular portion of the buccal mass bears bifurcated and short jaw rodlets (Fig. 7a, b). The radular formula of CASIZ 228939 is 37 × 19.1.19 (Fig. 7c). The rachidian tooth is triangular and short. The inner and outer surfaces of the inner lateral teeth have three denticles on each side of the central cusp (Fig. 7d). The central cusp on the inner lateral tooth is ~ 2× the length of the adjacent denticles. The middle lateral teeth have a short central cusp with three or four denticles (Fig. 7e). The outer lateral teeth have a rounded main cusp with three or four denticles (Fig. 7f).

**Figure 6.**
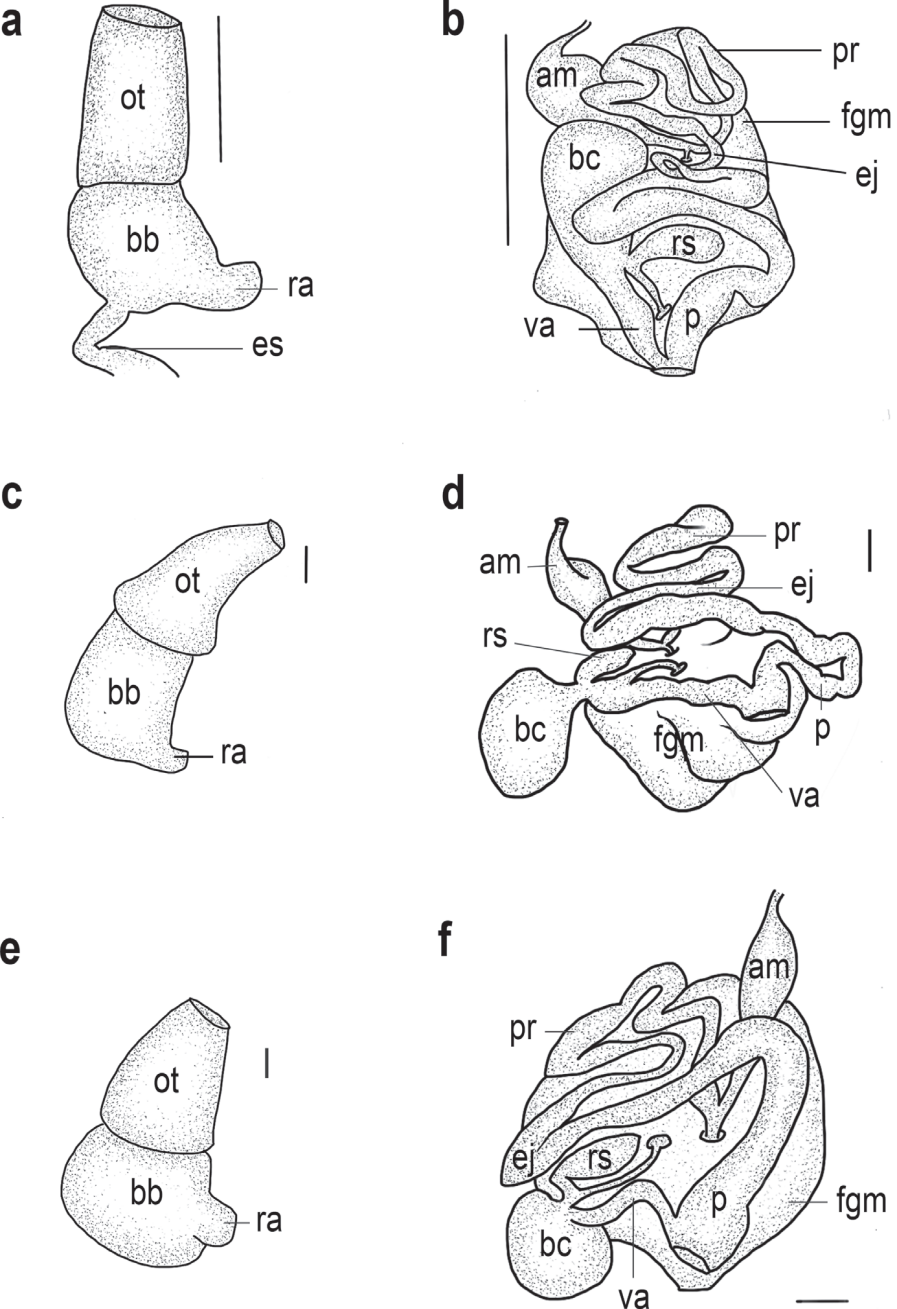
**a** buccal mass of *Goniobranchussinensis*, MISE-047-19 **b** reproductive system of *Goniobranchussinensis*, MISE-047-19 **c** buccal mass of *Goniobranchusverrieri*, CASIZ 203059 **d** reproductive system of *Goniobranchusverrieri*, CASIZ 203059 **e** bBuccal mass of *Goniobranchusfabulus* sp. nov., CASIZ 191271 **f** reproductive system of *Goniobranchusfabulus* sp. nov., CASIZ 191271. Abbreviations: am, ampulla; bb, buccal bulb; bc, bursa copulatrix; ej, ejaculatory duct; es, esophagus; fgm, female gland mass; ot, oral tube; p, penis; pr, prostate; ra, radular sac; rs, receptaculum seminis; va, vagina. Scale bars: 0.1 mm (**c, d, e, f**); 1 mm (**a, b**).

**Figure 7.**
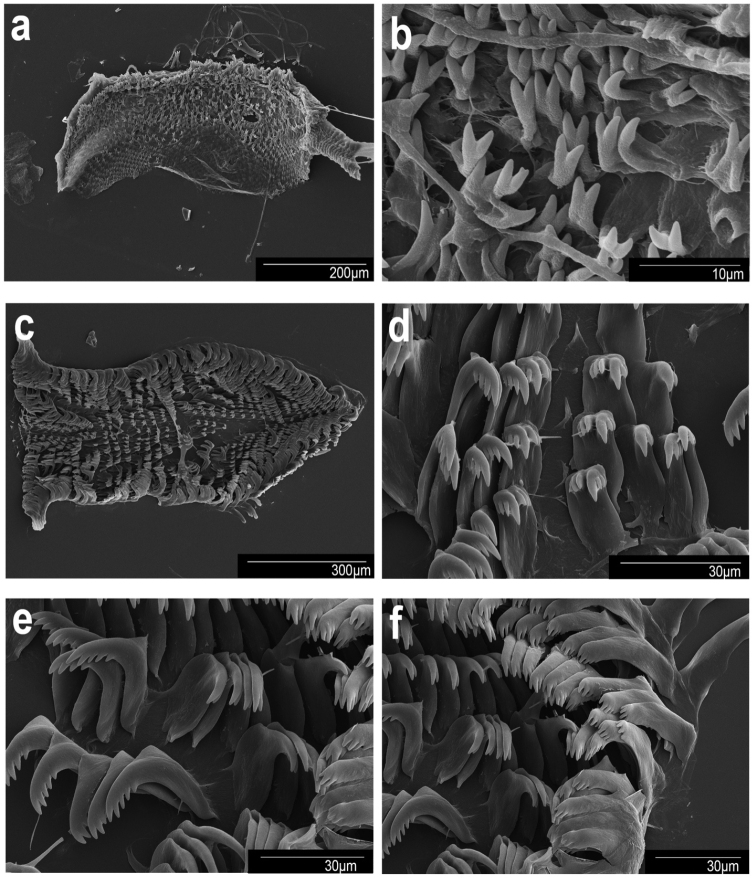
Scanning electron micrographs. *Goniobranchusalbonares*, CASIZ 228939, Philippines **a** jaw **b** jaw rodlets **c** radula **d** central teeth **e** mid-lateral teeth **f** outer lateral teeth.

***Reproductive system*** (Fig. 5b). The long, thick, tubular ampulla narrows into a diverging short oviduct and short vas deferens. The proximal prostatic portion of the vas deferens transitions into the muscular ejaculatory portion. The ejaculatory portion narrows and elongates into a wider, long, curved penial bulb that joins with the narrow distal end of the vagina. The vagina is elongate and narrow, joining the larger, spherical bursa copulatrix and the smaller, curved receptaculum seminis at its distal end. A moderately short uterine duct emerges from the receptaculum seminis, which is adjacent to the vagina, and enters into the female gland mass. The female gland mass has small albumen and membrane glands and a large mucous gland.

##### Remarks.

*Goniobranchusalbonares* was described by Rudman (1990) from New South Wales, Australia; he described the animal as having an elongate, ovate, opaque white mantle with a bright orange band on the edge of the mantle with the inside edge of the orange band being irregular. The rhinophores and gill branches were translucent white with opaque white edges, which is a distinctive feature of this species. Also, the notum was described as smooth, ringed by an orange marginal band and a yellow submarginal band. This morphological description matches well with the *G.albonares* specimens in this study, which are quite uniform in color pattern. The vas deferens in *G.albonares* is also shorter in comparison to that of all the other white *Goniobranchus* with marginal bands species included in this study. The phylogenetic tree also showed a fully supported (1/100%) monophyly for specimens (*n* = 4) of this species (intraspecific distance within *G.albonares* = 1.1–5.2%; Table 2).

*Goniobranchusalbonares* was included in this study together with all other white *Goniobranchus* with marginal bands based on Gosliner et al. (2018). However, in our concatenated phylogenetic tree, *G.albonares* is a sister clade to *G.collingwoodi*, *G.decorus*, *G.fidelis*, and *G.geminus*, and is genetically comparatively distant from the remainder of the white *Goniobranchus* species with marginal bands examined in this study (interspecific *p*-COI distances between *G.albonares* and *G.verrieri* = 16.0–18.2%; see Table 2). This suggests a case of convergent evolution of having a white body with marginal bands. Little is known about how predators perceive the color of the nudibranchs (as prey), which may provide clues to factors driving this remarkable similarity.

#### ﻿ Goniobranchus preciosus

Taxon classificationAnimaliaNudibranchiaChromodorididae

(Kelaart, 1858)

9AC9EFDA-7573-5669-8ED1-26104F1D4BD5

**Figure 8.**
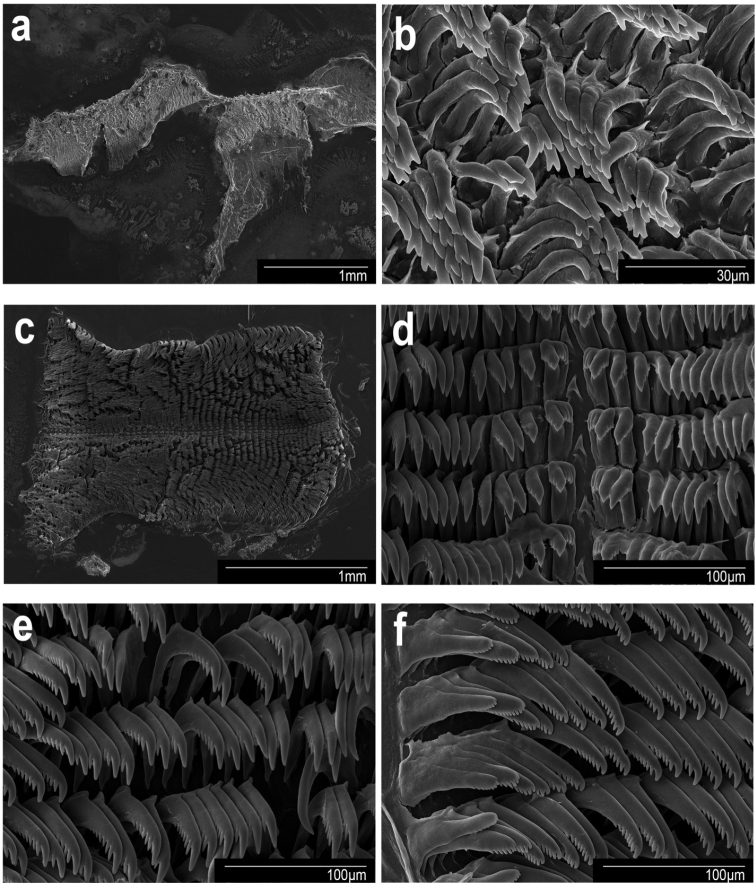
Scanning electron micrographs. *Goniobranchuspreciosus*, CASIZ 208574, Philippines **a** jaw **b** jaw rodlets **c** radula **d** central teeth **e** mid-lateral teeth **f** outer lateral teeth.

Figures 2c–f , 5c, d , 8a–f

Doris preciosa Kelaart, 1858: 98; 1883: 89.
Chromodoris preciosa : Eliot 1906: 642–643, pl. XLII, fig. 3; Eliot 1909: 92­­–93; Gosliner et al. 2008: 219, lower left and lower right photographs.
Goniobranchus preciosus : Gosliner et al. 2015: 222, lower left and lower right photographs; Gosliner et al. 2018: 152, lower left and lower right photographs.

##### Type locality.

Sri Lanka (as Ceylon), Indian Ocean.

##### Type material.

Most likely lost to science. Eliot (1906) refers to a few of Kelaart’s specimens being present in the collections of the Hancock Museum (now the Great North Museum) and that many of these specimens are useless for taxonomy. A search of the collections online indicates that no specimens of *Dorispreciosa* are currently held in their collection. We made comparisons to Kelaart’s original drawings and description (Kelaart, 1858), as well as to updates by Eliot (1906, 1909) and Rudman (1985).

##### Geographical distribution.

Widely distributed around the tropical and subtropical Indo-Pacific oceans (Rudman 1985; Debelius and Kuiter 2007; Coleman 2008; Gosliner et al. 2008, 2015, 2018) with specific reports from Sri Lanka (Kelaart 1858), west coast of India and the Andaman Islands (Kumar et al. 2019), Thailand (Mehrotra et al. 2021), Philippines, Indonesia, Malaysia (Gosliner et al. 2008), and Japan (Nakano 2018; Ono and Katou 2020). Records cited by Gosliner et al. (2008) from New Caledonia, Tonga, Fiji, Vanuatu, and Australia are of *Goniobranchusfabulus* sp. nov., not *G.preciosus*.

##### Material examined.

CASIZ 208420 (morphotype A), one specimen (10 mm preserved), subsampled for molecular data, sand slope with reef, 13.522°N, 120.947°E, Manila Channel, Puerto Galera, Oriental Mindoro Province, Mindoro, Philippines, 4–22 m depth, 11 April 2015, T.M. Gosliner 2015 Verde Island Passage Expedition. CASIZ 208415 (morphotype A), one specimen (9 mm preserved), subsampled for molecular data and dissected, School Beach, 13.517°N, 120.950°E, Batangas Channel, Puerto Galera, Oriental Mindoro Province, Mindoro, Philippines, 18 m depth, 10 April 2015, T.M. Gosliner 2015 Verde Island Passage Expedition. CASIZ 208574 (morphotype B), one specimen (11 mm preserved), subsampled for molecular data and dissected, School Beach, 13.516°N, 120.950°E, Batangas Channel, Puerto Galera, Oriental Mindoro Province, Mindoro, Philippines, 6–17 m depth, 8 April 2015, T.M. Gosliner 2015 Verde Island Passage Expedition. CASIZ 176752 (morphotype C), one specimen (10 mm preserved), subsampled for molecular data, Pulau Gut, 2.664°N, 104.167°E, Pulau Tioman, South China Sea, Peninsular Malaysia, 13 m depth, 4 October 2007, T.M. Gosliner. CASIZ 176761 (morphotype D), one specimen (9 mm preserved), subsampled for molecular data, Tiger Point, 2.889°N, 104.061°E, Pulau Tioman, South China Sea, Peninsular Malaysia, 17–19 m depth, 2 October 2007, T.M. Gosliner.

##### Description.

***External morphology*.** Living animal approximately 15 mm in length. Body white, with low tubercles on the notum; oval and elongated, with three marginal bands on the mantle edge. There is an outermost blue band followed by a deep red submarginal band and a yellow inner submarginal band. Brownish or orange dorsal spotting may be present over the surface of the mantle. In all cases the rhinophores are translucent reddish brown with white edges on the lamellae. The same pigment extends below the rhinophore club onto the stalks of the rhinophores. Rhinophore lamellae number 12–17. Gill branches reddish brown with white lines on the rachis. Nine or ten unipinnate gill branches held erectly when the gill is fully extended. This species exhibits four distinct morphotypes in addition to the unvarying elements described above. Morphotype A (Fig. 2c) has a translucent creamy white body with fine orange spots and blotches on the notum. The outermost portion of the mantle edge is surrounded by a thin opaque bluish white band, followed by a thicker deep red band and then a yellow-orange submarginal band. Gill and rhinophores are translucent red with white edges. Morphotype B (Fig. 2d) has a translucent pale yellow body with brown spots and blotches on the notum. The outermost portion of the mantle edge is surrounded by an opaque bluish white tinged band, followed by an irregular deep red and a yellow-orange submarginal band, with all three bands having similar widths. The gill and rhinophores are translucent brown with opaque cream edges. Morphotype C (Fig. 2e) has an opaque white body with a few low tubercles. The outermost portion of the mantle edge is surrounded by a thin, opaque, bluish white band, followed by thicker deep red and yellow-orange bands. The gill and rhinophores are translucent red with opaque white edges. Morphotype D (Fig. 2f) has a creamy white translucent body with densely speckled orange spots on the notum. The outermost portion of the mantle edge is surrounded by a thin opaque bluish white tinged band, followed by irregular deep red and yellow-orange bands, all three bands having similar widths. The gill and rhinophores are translucent red with opaque white edges.

***Buccal mass and radula (morphotype B)*.** The muscular portion of the buccal mass is ~ 2× the size of the oral tube length (Fig. 5c). The chitinous labial cuticle is found at the anterior end of the muscular portion of the buccal mass, bearing long, bifurcated jaw rodlets (Fig. 8a, b). The radular formula of CASIZ 208574 is 54 × 47.1.47 (Fig. 8c). The rachidian tooth has a flame-like shape and is blunt at the tips. The inner and outer surfaces of the inner lateral teeth have three or four denticles on each side of the central cusp (Fig. 8d). The central cusp on the inner lateral tooth is ~ 2× the length of the adjacent denticles. The middle lateral teeth have a long central cusp with 5–8 denticles (Fig. 8e). The outer lateral teeth are rounded and paddle-shaped with six or seven denticles (Fig. 8f).

***Reproductive system*** (Fig. 5d). The thick, tubular ampulla narrows into a diverging short oviduct and long vas deferens. The proximal prostatic portion of the vas deferens is narrow and convoluted, then transitions into an equally thin muscular ejaculatory portion. The narrow ejaculatory portion elongates into a wider section and again narrows prior to entering the short penial bulb, which joins with the distal end of the vagina. The vagina is short and moderately wide. It terminates at the junction of the large, spherical bursa copulatrix, the curved, pyriform receptaculum seminis, and the uterine duct. The long narrow uterine duct emerges from junction of the vagina, bursa copulatrix, and the receptaculum seminis and enters into the female gland mass. The female gland mass has small albumen and membrane glands and a large mucous gland.

##### Remarks.

Rudman (1985) redescribed specimens of *G.preciosus* from New Caledonia based on the description by Kelaart (1858) and the illustration in Eliot (1906) from Sri Lanka (as Ceylon). Rudman stated that Eliot’s (1906) reproduction of Kelaart’s drawing of *Dorispreci*os*a* did not match the original description of *G.preciosus* by Kelaart (1858). However, Kelaart’s written description and the reproduction of his drawing by Eliot (1906) clearly match the three main morphotypes of *G.preciosus* found in this study. Additionally, Eliot (1909) reported on another *G.preciosus* specimen collected by Willey in Sri Lanka that had a few obscure spots on its notum, but Eliot’s notes did not mention any light bluish tinge on the outermost mantle edge. Rudman (1985) doubted that Eliot’s (1909) specimen was the real *G.precio*s*us* due to these few obscure spots and the absence of a light bluish margin. Hence, Rudman (1985) considered his specimen from New Caledonia as *G.preciosus* based on the descriptions from both Kelaart and Eliot. However, Rudman’s specimen lacks the dense red spotting described by Kelaart, but illustrated by Eliot, and that is present in the specimens studied here. Eliot’s illustration matches *G.preciosus* morphotype A found in this study. Based on the phylogenetic data in this study, the morphotype that matches Kelaart’s description (morphotype D; Fig. 2f) and the morphotype that matched Rudman’s description (*G.fabulus* sp. nov.; Fig. 4a–c) are clearly distinct from each other. This distinction, as well as the fact that the species that Rudman identified as *G.preciosus* is not found in the Indian Ocean and appears to be restricted to the Western and Central Pacific, suggest separate species and Rudman’s *G.preciosus* is herein described as G.*fabulus* sp. nov. These species have been frequently confused and often considered as a single species (e.g., Gosliner et al. 2018), but there are clear morphological distinctions as found in this study. In *G.preciosus*, the mantle always has some low tubercles, whereas the notum is smooth in *G.fabulus*. The gill branches of *G.preciosus* are more erect than those of *G.fabulus*. The gill and rhinophores of *G.precious* are reddish brown, whereas they are reddish purple in *G.fabulus*. In *G.precious* the club and stalk of the rhinophores have reddish pigment whereas in *G.fabulus* only the rhinophore club is pigmented and the stalk is the same white as the body. The two species overlap in the Philippines (present study), but *G.preciosus* is found north and westwards from the Philippines and *G.fabulus* is found to the south and eastwards from there.

*Goniobranchuspreciosus* was recovered as a distinct species in the phylogenetic and ABGD analyses and was sister to a clade containing *G.daphne* (interspecific *p*-COI distances between *G.preciosus* and *G.daphne* = 7.4–7.9%; Table 2), *Goniobranchusfabulus* sp. nov. (interspecific *p*-COI distances between *G.preciosus* and *G.fabulus* sp. nov. = 6.8–9.2%; Table 2), and *G.sinensis* (interspecific *p*-COI distances between *G.preciosus* and *G.sinensis*.= 7.1–9.8%; Table 2). *Goniobranchuspreciosus* has a high level of intraspecific morphological diversity with the presence of four morphotypes confirmed in this study and yet showed little genetic difference (intraspecific distance within *G.preciosus* = 0.4–2.7%; Table 2). These four morphotypes have very close external morphological similarities with *G.verrieri* morphotype B and *G.sinensis*, with all of them having three marginal bands on the mantle edge and with *G.verrieri* morphotype B and some morphotypes of *G.sinensis* having spots and patches on the notum. However, *G.verrieri* morphotype B has a greatly reduced outer white band compared to the much wider bluish bands of *G.preciosus* and *G.sinensis*. Only very subtle external morphological differences separate *G.preciosus* from the other species in this study. *Goniobranchuspreciosus* morphotype A has a deeper red submarginal band while *G.verrieri* morphotype B has a paler red submarginal band. *Goniobranchuspreciosus* morphotype B has a pale yellow body coloration that was not observed in any other specimens in this study. *Goniobranchuspreciosus* morphotype C is very similar to *G.fabulus* morphotype A and *G.sinensis* morphotype C; however, the gill and rhinophore colors are not the same: *G.preciosus* has translucent red rhinophores and gills with opaque white edges, *G.fabulus* morphotype A has reddish purple rhinophores and gills with opaque white edges, and *G.sinensis* morphotype C has translucent red rhinophores and gills with opaque reddish purple edges. *Goniobranchuspreciosus* morphotype D has densely speckled orange spots on the notum and an opaque bluish white tinged band on the mantle edge and this character combination was not observed in any other specimens in this study. *Goniobranchuspreciosus* morphotype D also most closely matched the original external morphology of *G.preciosus* as described by Kelaart (1858).

With regards to internal morphology, *G.preciosus* and *G.sinensis* each have a flame-shaped rachidian tooth, but differ in their external colors and morphologies. *Goniobranchuspreciosus* has a tuberculate body texture, whereas *G.sinensis* has a smooth notum. The rhinophores of *G.preciosus* are reddish brown and have spots of the same color extending onto the rhinophoral stalk. In *G.sinensis*, the rhinophores have reddish purple edges along the lamellae of the club and solid reddish purple rather than scattered spots extending onto the rhinophore stalk. Both species have three marginal bands which are similar in color but in *G.preciosus* the innermost band is more yellow-orange whereas it is more yellow *in G.sinensis*. These differences in color are subtle but appear to be consistent in the specimens studied here.

The high morphological diversity of *G.preciosus* suggests two different forms of morphological adaptations. *Goniobranchuspreciosus* had different color patterns within the same locality, with two different morphotypes occurring both in the Philippines and in Peninsular Malaysia. At the same time, from a regional perspective, *G.preciosus* had color patterns specific to each locality. This is not the first time such a situation has been observed in nudibranchs, as previous studies have demonstrated a form of mimicry in chromodorid nudibranchs resulting in certain chromodorid species displaying morphological variation within a locality as well as individuals with same color pattern within the same locality turning out to be different species (Padula et al. 2016; Layton et al. 2018, 2020).

#### ﻿ Goniobranchus rubrocornutus

Taxon classificationAnimaliaNudibranchiaChromodorididae

(Rudman, 1985)

870E2DC2-E818-5670-9B72-4FBE029699CB

**Figure 9.**
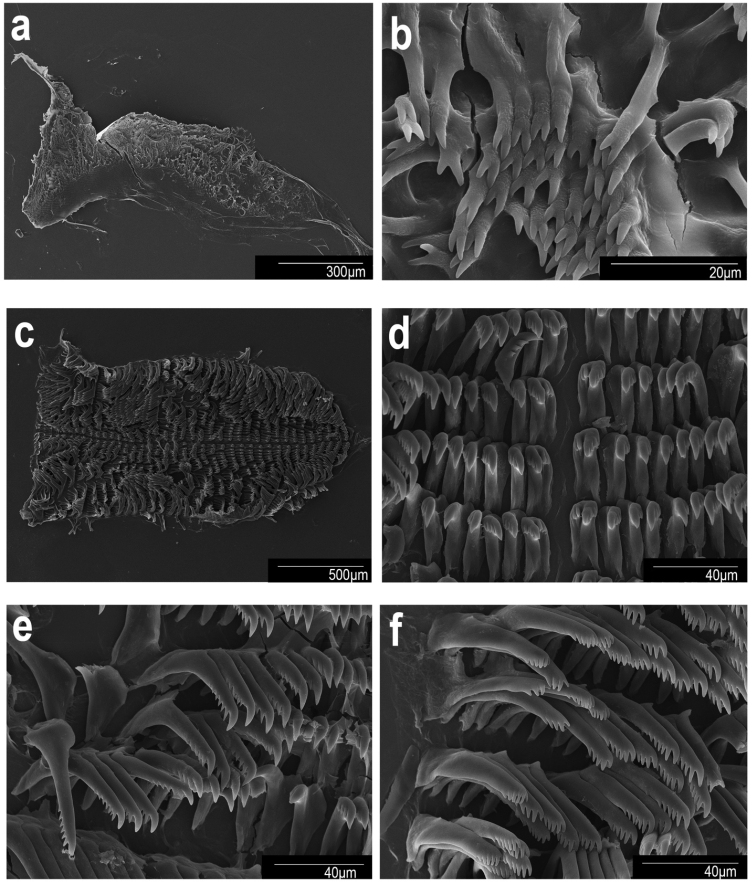
Scanning electron micrographs. *Goniobranchusrubrocornutus*, CASIZ 203047, Philippines. **a** jaw **b** jaw rodlets **c** radula **d** central teeth **e** mid-lateral teeth **f** outer lateral teeth.

Figures 2g, h , 5e, f , 9a–f

Glossodoris marginata (Pease, 1860): Baba 1938: 11–12 (misidentification).
Chromodoris rubrocornuta Rudman, 1985: 83, 283–286, figs 12F, 20A, 25, 26A; Gosliner et al. 2008: 221, bottom photograph.
Goniobranchus rubrocornutus : Gosliner et al. 2015: 224, middle right photograph; Gosliner et al. 2018: 154, middle right photograph.
Goniobranchus cf. albonares (Rudman, 1985): Mehrotra et al. 2021: 104, fig. 9l (misidentification).

##### Type locality.

Hong Kong.

##### Type material.

AM C138518, one specimen, Flynn Point, 22.467°N, 114.333°E, Hoi Ha, Hong Kong, China, depth not available, 18 April 1983, collector not available. Not examined in this study due to the original description in Rudman (1985) being sufficient for comparisons.

##### Geographical distribution.

Widely distributed around the tropical and subtropical Indo-Pacific oceans (Debelius and Kuiter 2007; Gosliner et al. 2008, 2015, 2018; Rudman 1985) with reports from Thailand (Mehrotra et al. 2021), Malaysia, Philippines, Hong Kong, Palau, American Samoa, Marshall Islands (Gosliner et al. 2008), Japan (Nakano 2018; Ono and Katou 2020), Australia (Rudman 1985), New Caledonia (Hervé 2010), and the Marianas Islands (Carlson and Hoff 2003).

##### Material examined.

CASIZ 203047 (morphotype A), one specimen (4 mm preserved), subsampled for molecular data and dissected, Verde Island Passage coast, 13.917°N, 120.617°E, Calatagan, Batangas Province, Luzon, Philippines, depth not available, 9 May 2014, T.M. Gosliner, 2014 Verde Island Passage Expedition. CASIZ 181235 (morphotype A), one specimen (4 mm preserved), dissected, Twin Rocks, 13.683°N, 120.883°E, Maricaban Strait, Mabini (Calumpan Peninsula), Batangas Province, Luzon, Philippines, depth not available, 22 May 2009, P. Paleracio, CAS Philippines Expedition May 2009. CASIZ 208563 (morphotype B), one specimen (3 mm preserved), subsampled for molecular data, School Beach, 13.516°N, 120.95°E, Batangas Channel, Puerto Galera, Oriental Mindoro Province, Mindoro, Philippines, 6–18 m depth, 13 April 2015, T.M. Gosliner, 2015 Verde Island Passage Expedition.

##### Description.

***External morphology*.** Length of living animal 7–14mm. Body oval and elongated, with two marginal bands on the mantle edge. Six to nine unipinnate gill branches, 8–14 lamellae on rhinophores. The color patterns of this species can be divided into two distinct morphotypes. Morphotype A (Fig. 2g) has a translucent creamy white body. The outermost portion of the mantle edge is surrounded by an orange band, followed by an irregular red band, followed by another irregular opaque white band. Gill branches and rhinophores are translucent, deep red with either red or white edges. Morphotype B (Fig. 2h) has an opaque white body. The outermost portion of the mantle edge is surrounded by a red band, followed by a yellow submarginal band and both bands have similar widths. The gill and rhinophores are translucent deep red with bluish white tinged edges.

***Buccal mass and radula*.** The muscular portion of the buccal mass approximately the same size as the oral tube length (Fig. 5e). The chitinous labial cuticle is found at the anterior end of the muscular portion of the buccal mass and bears bifurcated and short jaw rodlets (Fig. 9a, b). The radular formula of CASIZ 181235 is 39 × 27.1.27 (Fig. 9c). The rachidian tooth is thin and linear. The inner and outer surface of the inner lateral teeth have two or three denticles on each side of the central cusp (Fig. 9d). The central cusp on the inner lateral tooth is ~ 2× the length of the adjacent denticles. The middle lateral teeth have a short central cusp with 5–7 denticles (Fig. 9e). The outer lateral teeth have a rounded main cusp with 3–5 denticles (Fig. 9f).

***Reproductive system*** (Fig. 5f). The thick, tubular ampulla narrows into a diverging short oviduct and long vas deferens. The proximal prostatic portion of the vas deferensis thin and convoluted and transitions into the muscular ejaculatory portion. The long, narrow, convoluted ejaculatory portion transitions into a wider, long penial bulb, which joins with the distal end of the vagina. The vagina is proximally narrow and elongated, transitions into a larger, spherical bursa copulatrix and large receptaculum seminis at its distal end. A moderately long uterine duct emerges from this junction of vagina, bursa copulatrix, and receptaculum seminis. The uterine duct connects the receptaculum seminis with the female gland mass. The female gland mass has smaller albumen and membrane glands and a larger mucous gland.

##### Remarks.

In this study, *G.rubrocornutus* morphotype A matched with Rudman’s (1985)*G.rubrocornutus* from Hong Kong: a creamy white translucent body with the outermost portion of the mantle edge surrounded by an orange band, followed by an irregular red band and an irregular opaque white band. The gill branches and rhinophores were translucent deep red with either red or white edges. *Goniobranchusrubrocornutus* morphotype B only has two marginal bands with the outermost red band followed by a yellow submarginal band, and this pattern does not match with Rudman’s description of *G.rubrocornutus*. In this case the inner white submarginal band may simply be masked by the opaque white body color of morphotype B rather than the cream body color of morphotype A. However, in our phylogenetic and species delimitation analyses, *G.rubrocornutus* morphotype B was clustered together with morphotype A and both morphotypes did not show any genetic differences (uncorrected pairwise distance = 0.0%). Thus, morphotype B very likely represents a different color variation of *G.rubrocornutus*. Recently, molecular work has revealed the presence of mimicry adaptation in chromodorid nudibranchs (e.g., Padula et al. 2016; Layton et al. 2018). Sympatric specimens of chromodorid nudibranchs with different color patterns were found to be the same species (Layton et al. 2018), and this is also the case with our *G.rubrocornutus* morphotypes, where both morphotypes are sympatric. In this case, these variations are not likely different cases of mimicry, but simply color variants. Despite the variations observed here, few records of this species have been misidentified, with the exception of Mehrotra et al. (2021), where *G.rubrocornutus* was identified as G.cf.albonares. The specimen illustrated clearly has red rhinophores with white edging rather than white rhinophores and the orange, red, and opaque white marginal and submarginal bands that are characteristic of *G.rubrocornutus*.

#### ﻿ Goniobranchus sinensis

Taxon classificationAnimaliaNudibranchiaChromodorididae

(Rudman, 1985)

8E42DAE8-2FA4-5DE4-9127-E2D1157AF69D

**Figure 10.**
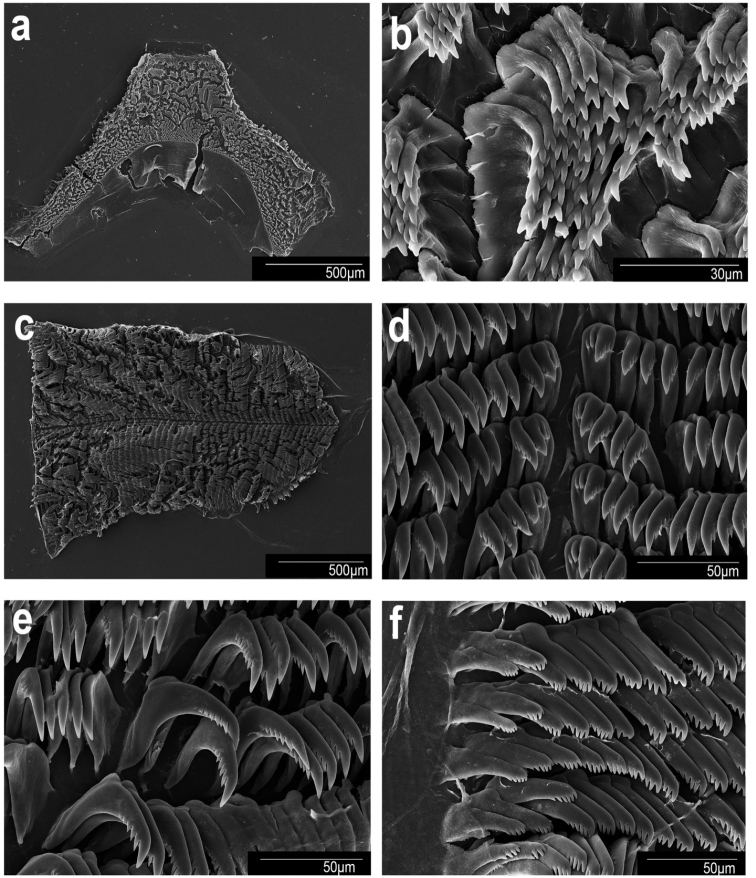
Scanning electron micrographs. *Goniobranchussinensis*, MISE-047-19, Kagoshima, Japan. **a** jaw **b** jaw rodlets **c** radula **d** central teeth **e** mid-lateral teeth **f** outer lateral teeth.

Figures 3a–d , 6a, b , 10a–f

Glossodoris marginata (Pease, 1860): Baba 1938: 11–12, fig. 8; Abe 1964: 47, pl. 21, fig. 74; Lin and Tchang 1965: 10, pl. 1, fig. 11 (misidentifications).
Chromodoris marginata (Pease, 1860): Orr 1981: 27 (misidentification)
Chromodoris sinensis Rudman, 1985: 83, 272–275, figs 12C, 13C, 14C, 15C, 18, 19; Gosliner et al. 2008: 219, bottom photograph.
Goniobranchus sinensis : Gosliner et al. 2015: 223, middle left photograph; Gosliner et al. 2018: 153, middle left photograph.

##### Type locality.

Hong Kong.

##### Type material.

AM C139295, one specimen, Fan Tsang Chau Island, 22.367°N, 114.400°E, Hong Kong, China, 10 m depth, 11 August 1983. Type material not examined due to high level of detailed work provided by the original description in Rudman (1985).

##### Geographical distribution.

This species appears to be restricted to areas of the southeast Asian mainland and the islands of Japan, Taiwan, and islands off eastern Peninsular Malaysia (Debelius and Kuiter 2007; Coleman 2008; Gosliner et al. 2008, 2015, 2018) with reports from the Andaman Islands (Kumar et al. 2019), the east coast of Thailand (Mehrotra et al. 2021), the east coast of Peninsular Malaysia (present study), Japan (Nakano 2018; Ono and Katou 2020), Taiwan (Jie et al. 2009), Hong Kong (Rudman 1985), and the Gulf of Oman (Fatemi and Attaran-Fariman 2015).

##### Material examined.

MISE-047-19 (morphotype A), one specimen, subsampled for molecular data and dissected, 31.281°N, 130.203°E, Kagoshima, Japan, 10 m depth, 14 July 2019, A. Tsuyuki. MISE-037-19 (morphotype A), one specimen, subsampled for molecular data, Sakurajima Evacuation Port Number 4, 31.552°N, 130.632°E, Kagoshima, Japan, 10 m depth, 10 July 2019, H. Kise. MISE-039-19 (morphotype A), one specimen, subsampled for molecular data, east side of Okiko-jima, 31.544°N, 130.617°E, Kagoshima, Japan, 8 m depth, 12 July 2019, G.Y. Soong. MISE-010-19 (morphotype B), one specimen, subsampled for molecular data and dissected, Tengan, 26.400°N, 127.833°E, Okinawa-jima Island, Japan, 8 m depth, 3 May 2019, G.Y. Soong. MISE-056-19 (morphotype B), one specimen, subsampled for molecular data, Tengan, 26.400°N, 127.833°E, Okinawa-jima Island, Japan, 12 m depth, 27 October 2019, G.Y. Soong. MISE-024-18 (morphotype B), one specimen, subsampled for molecular data, Tengan, 26.400°N, 127.833°E, Okinawa-jima Island, Japan, 7 m depth, 12 April 2018, G.Y. Soong. MISE-024-19 (morphotype B), one specimen, subsampled for molecular data, Tengan, 26.400°N, 127.833°E, Okinawa-jima Island, Japan, 5 m depth, 16 June 2019, Y. Kushida. MISE-009-19 (morphotype B), one specimen, subsampled for molecular data, Tengan, 26.400°N, 127.833°E, Okinawa-jima Island, Japan, 8 m depth, 3 May 2019, G.Y. Soong. MISE-055-19 (morphotype B), one specimen, subsampled for molecular data, Tengan, 26.400°N, 127.833°E, Okinawa-jima Island, Japan, 8 m depth, 27 October 2019, H. Kise. MISE-020-18 (morphotype B), one specimen, subsampled for molecular data, Tengan, 26.400°N, 127.833°E, Okinawa-jima Island, Japan, 9 m depth, 12 April 2018, G.Y. Soong. MISE-010-19 (morphotype B), one specimen, subsampled for molecular data, Tengan, 26.400°N, 127.833°E, Okinawa-jima Island, Japan, 8 m depth, 3 May 2019, G.Y. Soong. MISE-023-18 (morphotype B), one specimen, subsampled for molecular data, Tengan, 26.400°N, 127.833°E, Okinawa-jima Island, Japan, 7 m depth, 12 April 2018, G.Y. Soong. MISE-018-19 (morphotype B), one specimen, subsampled for molecular data, Red Beach, 26.447°N, 127.912°E, Okinawa-jima Island, Japan, 6 m depth, 19 May 2019, G.Y. Soong. MISE-022-18 (morphotype B), one specimen, subsampled for molecular data, Tengan, 26.400°N, 127.833°E, Okinawa-jima Island, Japan, 10 m depth, 12 April 2018, G.Y. Soong. MISE-008-19 (morphotype B), one specimen, subsampled for molecular data, Tengan, 26.400°N, 127.833°E, Okinawa-jima Island, Japan, 8 m depth, 3 May 2019, G.Y. Soong. CASIZ 176759 (morphotype C), one specimen, subsampled for molecular data, Waterfall Bay, 2.720°N, 104.195°E, Pulau Tioman, South China Sea, Peninsular Malaysia, 14 m depth, 4 October 2007, T.M. Gosliner et al. CASIZ 175727 (morphotype C), one specimen (2 mm preserved), subsampled for molecular data, Pulau Gut, 2.664°N, 104.167°E, Pulau Tioman, South China Sea, Peninsular Malaysia. 14 m depth, 4 October 2007, T.M. Gosliner. CASIZ 189457 (morphotype C), one specimen (3 mm preserved), subsampled for molecular data, location not available, GPS data not available, Peninsular Malaysia, depth not available, 4 October 2007, T.M. Gosliner.

##### Description.

***External morphology*.** Living animal ~ 10 mm in length. Body smooth, without tubercles, oval and elongated, with three marginal bands on the mantle edge. Seven to ten unipinnate gill branches, 13–18 rhinophore lamellae. The species has three distinct morphotypes based on color patterns. Morphotype A (Fig. 3a) has a translucent creamy white body with no spots on the notum. The outermost portion of the mantle edge is surrounded by a thin whitish blue band, followed by one each of thicker red and yellow bands. The gill and rhinophores are translucent red with reddish purple edges. Morphotype B (Fig. 3b, c) has a translucent white body with brown spots on the notum. The outermost portion of the mantle edge is surrounded by an opaque bluish white tinged band, followed by red and yellow submarginal bands, and all three bands have similar widths. The gill and rhinophores are translucent red with opaque white edges. Morphotype C (Fig. 3d) has a creamy white but translucent body with fine orange spots on the notum. The outermost portion of the mantle edge is surrounded by a thin opaque bluish white tinged band, followed by a thicker irregular red band, and then a yellow submarginal band of similar thickness to the red band. Gill and rhinophores are translucent red with reddish purple edges.

***Buccal mass and radula (morphotype A)*.** The muscular portion of the buccal mass approximately the same size as the oral tube length (Fig. 6a). The chitinous labial cuticle found at the anterior end of the muscular portion of the buccal mass bearing bifurcated and long jaw rodlets (Fig. 10a, b). The radular formula of MISE-010-19 and MISE-047-19 (Fig. 10c) are 46 × 40.1.40 and 52 × 40.1.40, respectively. The rachidian tooth is triangular, thin, with a blunt tip. The innermost lateral teeth have two or three denticles on the inner side and 3–5 denticles on the outer side of the central cusp (Fig. 10d). The central cusp on the inner lateral tooth is elongate and ~ 2× the length of the adjacent denticles. The middle lateral teeth have a short central cusp with six or seven denticles (Fig. 10e). The outer lateral teeth have a rounded main cusp with five denticles (Fig. 10f).

***Reproductive system*** (Fig. 6b). The thick, tubular ampulla narrows into a diverging short oviduct and long vas deferens. The proximal prostatic portion of the vas deferens is thin and convoluted and transitions into the muscular ejaculatory portion. The long, narrow, convoluted ejaculatory portion transitions into a wider and long curved penial bulb, which joins with the distal end of the vagina. The vagina is narrow and elongated and transitions into a larger, spherical bursa copulatrix and the smaller, curved receptaculum seminis at its distal end. A moderately long uterine duct emerges from this junction of vagina, bursa, and receptaculum seminis. The uterine duct connects the receptaculum seminis with the female gland mass. The female gland mass has smaller albumen and membrane glands and a larger mucous gland.

##### Remarks.

Our *G.sinensis* morphotype A specimens are the same as Rudman’s (1985) specimens; all of Rudman’s (1985) specimens were collected from Hong Kong. He only found one morphotype, with a translucent creamy white body and the outermost portion of the mantle edge surrounded by a thin white band, followed by one each of thicker red and yellow bands. The gill and rhinophores were translucent red with reddish purple edges. Some of the specimens he collected also had fine orange-brown specks on the notum; however, this morphological trait was observed in comparatively few of the newly collected specimens and is found in morphotype C (Fig. 3d). Rudman (1985) also synonymized specimens documented by Baba (1938) and Abe (1964) from Japan as *G.sinensis*, further supporting the identification of our specimens from Kagoshima, Japan as *G.sinensis*. Morphotype A has thus been reported from Hong Kong and Japan. In our study, we also observed two more morphotypes of *G.sinensis*: morphotype B from Okinawa, Japan and morphotype C from Peninsular Malaysia.

*Goniobranchussinensis* demonstrates intraspecific variation (intraspecific *p*-COI distance within *G.sinensis* = 0.0–1.4%) in morphology based on geographic location, with specimens collected from Peninsular Malaysia, Okinawa, and mainland Japan in this study. Body patterns of nudibranchs can vary depending on environmental factors (Rudman 1991), and this may explain the morphological variation in *G.sinensis* as observed by Rudman (1991) and in the current study. Distinctive features of the external morphology are included in the remarks for *G.preciosus*, the species with which this species has been most frequently confused.

#### ﻿ Goniobranchus verrieri

Taxon classificationAnimaliaNudibranchiaChromodorididae

(Crosse, 1875)

9C937852-9C5B-515B-805E-6DF7BD8445AD

**Figure 11.**
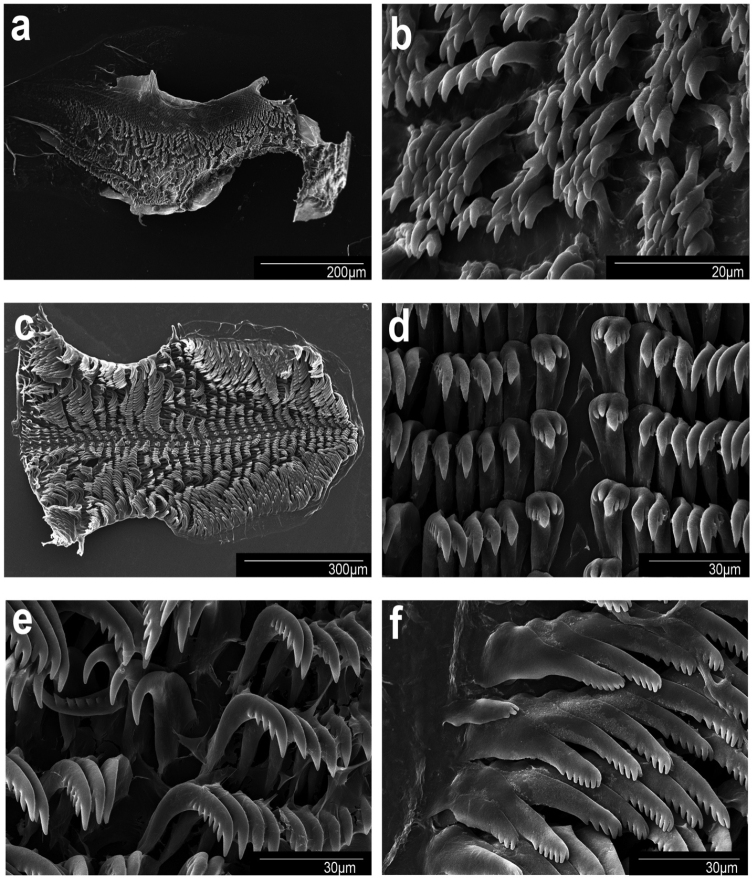
Scanning electron micrographs. *Goniobranchusverrieri*, CASIZ 203059, Philippines. **a** jaw **b** jaw rodlets **c** radula **d** central teeth **e** mid-lateral teeth **f** outer lateral teeth.

Figures 3e, f , 6c, d , 11a–f

Doris marginata Pease, 1860: 30 (junior homonym of both Dorismarginata Montagu, 1804: 79 and Dorismarginata Quoy & Gaimard, 1832: 255–256).
Goniodoris verrieri Crosse, 1875: 313, 314, pl. 12, fig. 5.
Chromodoris marginata : Bergh, 1880: 27, pl. 13, figs 22, 23; Risbec 1928: 133–136, fig. 33, pl. 6, fig. 4; Risbec 1953: 63–66, fig. 26; Kay 1979: 467, 468, fig. 150D.
Glossodoris verrieri : Pruvot-Fol 1951: 155.
Chromodoris verrieri : Risbec 1953: 80; Rudman 1985: 262–267, figs 12A, 13A, 14, 15A; Gosliner et al. 2008: 221, top photograph.
Chromodoris trimarginata (Winckworth, 1946): Kay and Young 1969: 205, 206, figs 45, 55 (misidentification).
Goniobranchus verrieri : Gosliner et al. 2015: 223, top right photograph; Gosliner et al. 2018: 153, top right photograph.
Chromodoris sinensis Rudman, 1985: 263, fig. 12C; Yonow 2001: 26, pl 3, fig. 6 (misidentifications).

##### Type locality.

Noumea, New Caledonia.

##### Type material.

Most likely lost to science. Crosse’s types are deposited in the Muséum national d’Histoire naturelle (Paris), but the list of types by Valdés and Heros (1998) of Recent and fossil opisthobranchs does not mention any material of *Goniodorisverrieri* Crosse, 1875. We base our identification from Crosse’s illustration (1875: pl. 12, fig. 5), which agrees with the morphological study of Rudman (1985).

##### Geographical distribution.

Widely distributed around the tropical and subtropical Indo-Pacific oceans (Rudman 1985; Debelius 1996; Debelius and Kuiter 2007; Coleman 2008; Gosliner et al. 2008, 2015, 2018) with reports from across South Africa, Madagascar, Indonesia, Papua New Guinea, Philippines, Midway Atoll, Hawaiian Islands (Gosliner et al. 2018), Australia (Slack-Smith and Bryce 2004; Nimbs and Smith 2016), Tanzania (Rudman 1985), Thailand (Mehrotra et al. 2021), Mozambique (Strömvoll and Jones 2019), Japan (Nakano 2018; Ono and Katou 2020), Taiwan (Jie et al. 2009), New Caledonia (Hervé 2010), Marshall Islands (Rudman 1985), and Mariana Islands (Carlson and Hoff 2003).

##### Material examined.

CASIZ 203059 (morphotype A), one specimen (3 mm preserved), subsampled for molecular data and dissected, Balibago dive site, 13.932°N, 120.611°E., Verde Island Passage Coast, Calatagan, Batangas Province, Luzon Island, Philippines, 12 m depth, 17 May 2014, S. Matsuda, 2014 Verde Island Passage Expedition. CASIZ 208442 (morphotype B), one specimen (5 mm preserved), subsampled for molecular data and dissected. Culebra (Bonito) Island, 13.617°N, 120.933°E, Maricaban Island, Tingloy, Batangas Province, Luzon, Philippines, 3–30 m depth, 18 April 2015, G. Paulay, 2015 Verde Island Passage Expedition.

##### Description.

***External morphology*.** Living animals approximately 11–17 mm in length. Body oval, with two marginal bands of similar widths on the mantle edge. Gill and rhinophores are translucent red with a mix of red and white edges. Four to eight unipinnate gill branches. Ten or eleven lamellae on rhinophores. The color patterns of this species can be divided into two distinct morphotypes. Morphotype A (Fig. 3e) has an opaque white body. The outermost portion of the mantle edge is surrounded by a red margin and a yellow submarginal band with both bands of similar widths. Morphotype B (Fig. 3f) has a translucent creamy white body with small orange spots on the notum. The outermost portion of the mantle edge is surrounded by a very thin opaque white band, followed by a red and a yellow submarginal band.

***Buccal mass and radula (morphotype A)*.** The muscular portion of the buccal mass is approximately the same size as the oral tube length (Fig. 6c). The chitinous labial cuticle found at the anterior end of the muscular portion of the buccal mass bearing bifurcated and short jaw rodlets (Fig. 11a, b). The radular formula of CASIZ 203059 is 37 × 28.1.28 (Fig. 11c). The rachidian tooth is flame-like in shape and short. The inner and outer surfaces of the inner lateral teeth have three denticles on each side (Fig. 11d). The central cusp on the inner lateral tooth is ~ 2× the length of the adjacent denticles. The middle lateral teeth have a short central cusp with approximately four or five denticles (Fig. 11e). The outer lateral teeth have a rounded tooth shaped with ~ 2–4 denticles (Fig. 11f).

***Reproductive system*** (Fig. 6d). The thick, tubular ampulla narrows into a diverging short oviduct and long vas deferens. The proximal prostatic portion of the vas deferens is wide and convoluted and transitions into the muscular ejaculatory portion. The long, narrow, convoluted ejaculatory portion transitions into a wider, long penial bulb, which joins with the distal end of the vagina. The thick muscular vagina is elongated and transitions into a larger, spherical bursa copulatrix. At this junction of the vagina and bursa copulatrix, the smaller pyriform receptaculum seminis also connects. The moderately long uterine duct that emerges from the junction of the vagina, bursa copulatrix, and receptaculum seminis enters into the female gland mass. This uterine duct junction also extends proximally on one side and includes a larger portion of the vagina. The female gland mass has small albumen and membrane glands and a large mucous gland.

##### Remarks.

*Goniobranchusverrieri* was originally described by Crosse (1875) from New Caledonia. The species had been previously described by Pease (1860) as *Dorismarginata* from Hawaiʻi. However, the name *Dorismarginata* was pre-occupied: several different species had been given the same name and *Goniobranchusverrieri* is the next available name for this species. Crosse described the animal as having a white body and the mantle edged in a light red margin and a yellow tinged submarginal band. This description matches the external morphology of the *G.verrieri* morphotype A in this study and specimens studied by Rudman (1985).

*Goniobranchusverrieri* morphotype B has a creamy translucent body with small orange spots on the notum and three marginal bands on mantle edge. Although this pattern did not match with the original description of *G.verrieri*, the phylogenetic and species delimitation analyses in this study showed that *G.verrieri* morphotype B is clustered with morphotype A. Based on this result, we consider morphotype B a color variation of *G.verrieri*. Both morphotypes also showed little genetic differences (intraspecific *p*-COI distance within *G.verrieri* = 1.3–3.7%), also suggesting that *G.verrieri* has morphological variation, similarly observed in some other white *Goniobranchus* species with marginal bands in this study. The vast majority of specimens of *G.verrieri* closely resemble morphotype A and there has been relatively little confusion of this species with others that have a white body and marginal bands. Spotted specimens of *G.verrieri* could be confused with *G.preciosus*, but have a more spreading gill plume whereas *G.preciosus* always have an erect gill plume.

#### ﻿ Goniobranchus fabulus

Taxon classificationAnimaliaNudibranchiaChromodorididae

Soong & Gosliner sp. nov.

8BA70376-3275-5746-8EA5-C6E95BB4E707

http://zoobank.org/A8690AEB-E87C-4F2D-985F-98404B87644A

**Figure 12.**
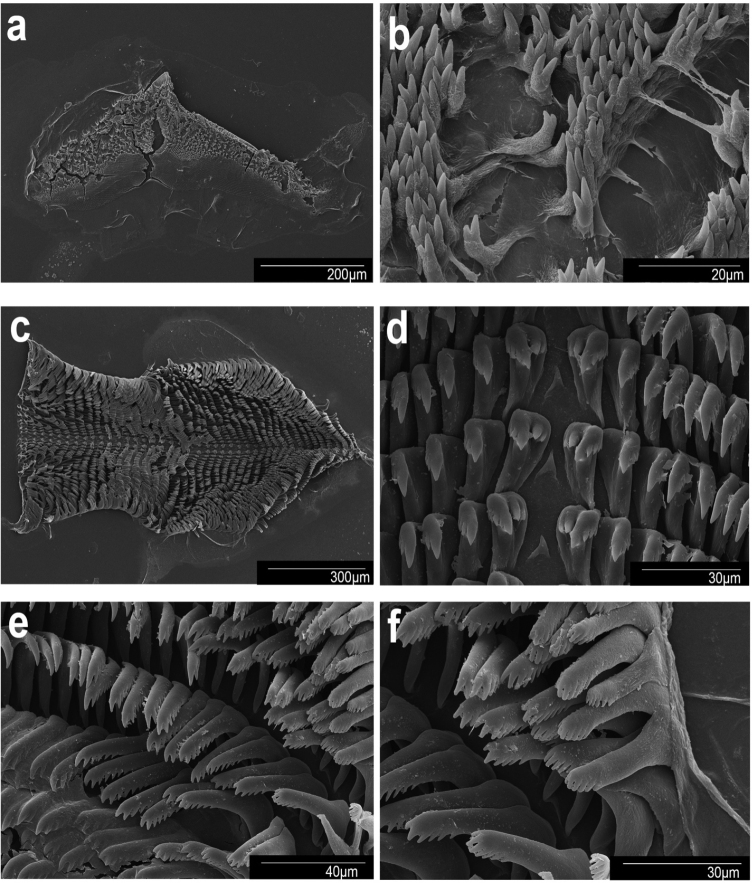
Scanning electron micrographs. *Goniobranchusfabulus* sp. nov., CASIZ 191271, Philippines. **a** jaw **b** jaw rodlets **c** radula **d** central teeth **e** mid-lateral teeth **f** outer lateral teeth.

Figures 4a–d , 6e, f , 12a–f

Chromodoris preciosa (Kelaart, 1858): Rudman 1985: figs 12b, 13b, 17; Gosliner et al. 2008: 219, upper right photo (misidentifications).
Goniobranchus preciosus (Kelaart, 1858): Gosliner et al. 2015: 222, lower middle right photo; Gosliner et al. 2018: 152: middle right photo (misidentifications).

##### Type material.

***Holotype***: CASIZ 191271 (morphotype B), one specimen (5 mm preserved), subsampled for molecular data and dissected. Siar Island, 5.187°S, 145.807°E, Madang Province, Papua New Guinea, depth not available, 16 November 2012, V. Knutson, Papua New Guinea Biodiversity Expedition 2012.

***Paratypes***: CASIZ 177517 (morphotype A), one specimen (3 mm preserved), subsampled for molecular data, Arthur’s Rock, 13.417°N, 120.517°E, Maricaban Strait, Mabini (Calumpan Peninsula), Batangas Province, Luzon, Philippines, 3 m depth, 21 March 2008, T.M. Gosliner et al., Philippines Expedition March 2008. CASIZ 177685 (morphotype A), one specimen (6 mm preserved), subsampled for molecular data, Bethlehem Channel, 13.672°N, 120.841°E, Bethlehem, Maricaban Island, Batangas Province, Philippines, 15 m depth, 20 April 2008, T.M. Gosliner. CASIZ 201949 (morphotype A), one specimen (5 mm preserved), subsampled for molecular data, Lago de Oro Hotel, 13.917°N, 120.616°E, Verde Island Passage coast, Calatagan, Batangas Province, Luzon Island, Philippines, 2 m depth, 19 May 2014, VIP Team, 2014 Verde Island Passage Expedition. CASIZ 191118 (morphotype B), one specimen (4 mm preserved), subsampled for molecular data, Mangroves, GPS, Madang Province, Papua New Guinea, 3 m depth, 10 November 2012, Papua New Guinea Biodiversity Expedition 2012.

##### Geographical distribution.

This species appears to be restricted to the western and southern central Pacific tropics (Gosliner et al. 2008, 2015, 2018) with reports from the Philippines (present study), Japan (Nakano 2018), Papua New Guinea, New Caledonia, Tonga, Vanuatu (Gosliner et al. 2008), Australia, and Fiji (Rudman 1985).

##### Description.

***External morphology*.** Living animals 12–18 mm in length. Body oval with three marginal bands on the mantle edge. Notum smooth with no apparent spots. Six to ten unipinnate gill branches. Eleven or twelve lamellae on rhinophores. The color pattern exhibits two distinct morphotypes. Morphotype A (Fig. 4a–c) has a creamy opaque white body. The outermost portion of the mantle edge is tinged an opaque bluish white, followed by a deep red band, followed by a yellow submarginal band, and then an opaque white band, with all bands having similar widths. Gill branches and rhinophores are reddish purple with white edges. Morphotype B (Fig. 4d) has an opaque creamy white body. The outermost portion of the mantle edge is surrounded by a speckled opaque white band, followed by a deep red band, a yellow submarginal band, and then an innermost opaque white band. The gill and rhinophores are reddish purple with white edges and opaque white speckles.

***Buccal mass and radula (morphotype B)*.** The muscular portion of the buccal mass is approximately the same size as the oral tube length (Fig. 6e). The chitinous labial cuticle found at the anterior end of the muscular portion of the buccal mass and bears bifurcated and short jaw rodlets (Fig. 12a, b). The radular formula of CASIZ 191271 is 42 × 35.1.35 (Fig. 12c). The rachidian tooth is triangular. The innermost lateral teeth have two denticles on the inner side of the cusp and three or four denticles on the outer side (Fig. 12d). The central cusp on the inner lateral tooth is elongate and ~ 2× the length of the adjacent denticles. The middle lateral teeth have an elongated central cusp with 5–7 denticles (Fig. 12e). The outer lateral teeth have a rounded tooth with 2–5 denticles (Fig. 12f).

***Reproductive system*** (Fig. 6f). The thin, tubular ampulla narrows into a diverging short oviduct and long vas deferens. The proximal prostatic portion of the vas deferens is thin and convoluted and transitions into the muscular ejaculatory portion. The long, narrow, convoluted ejaculatory portion transitions into a wider, long penial bulb, which joins with the moderately wide distal end of the vagina. The vagina is elongate and narrow, joining the larger, spherical bursa copulatrix and the smaller, curved receptaculum seminis at its distal end. A moderately long uterine duct that emerges from this junction of vagina, bursa copulatrix, and receptaculum seminis. The uterine duct connects the receptaculum seminis with the female gland mass. The female gland mass has smaller albumen and membrane glands and a larger mucous gland.

##### Etymology.

*Goniobranchusfabulus* sp. nov. is named after the Latin word which, in one translation, means a small bean, in reference to the body shape of the nudibranch.

##### Remarks.

*Goniobranchusfabulus* sp. nov. was recovered as a sister species to *G.daphne* in our phylogenetic analyses, with an interspecific distance of 2.5–4.5% (Table 2). *Goniobranchusdaphne* possess red spots of different sizes on the notum and can only be found in the Australian waters.

*Goniobranchusfabulus* sp. nov. morphotype A in our study matches well with Rudman’s (1985) description of *Goniobranchuspreciosus* from New Caledonia based on morphological characteristics. However, in our opinion the morphological characteristics of *G.preciosus* sensu Rudman did not match with the original description of *G.preciosus* and our specimen sequences are also genetically distinct from *G.preciosus* in this study (interspecific *p*-COI distance between *G.fabulus* and *G.preciosus* = 6.8–9.2%) (Fig. 1; Table 2). Hence, we have assigned *G.preciosus* sensu Rudman (1985) to *G.fabulus* sp. nov.

*Goniobranchusfabulus* sp. nov. morphotype B is slightly different from morphotype A in having opaque white speckles all over the gills and around the outermost edge of the mantle. This morphotype is only known from Papua New Guinea (Wakeling 2001; Gosliner et al. 2018). There is little genetic difference between the two morphotypes (intraspecific *p*-COI distances within *G.fabulus* sp. nov. = 0.2–3.4%). Confusion of this species with *G.preciosus* is discussed in the remarks section of *G.preciosus*.

## ﻿Discussion

*Goniobranchusfabulus* sp. nov. is known from the Philippines south and eastwards to Australia, Fiji, and Tonga. Many of the other species in this study are found in the Coral Triangle with overlap specifically in the Philippines; however, due to different geographical distributions, morphological differences, and the addition of new molecular data from this study, the six species examined here can be considered distinct.

As with other groups within Chromodorididae, the results of this study show that white *Goniobranchus* species with various marginal bands can be difficult to accurately identify based solely on external morphology due to similar color patterns. Although color pattern differences were distinct between species in this study, color pattern variations within species were also observed. In our study, *G.verrieri*, *G.preciosus*, *G.rubrocornutus*, *G.sinensis*, and *G.fabulus* sp. nov. displayed color polymorphism. Previous studies on chromodorid nudibranchs have also confirmed polymorphism (Padula et al. 2016; Layton et al. 2018, 2020), hypothesized to be due to Müllerian mimicry in which the nudibranchs mimic one another as protection from predators (Rudman 1991; Cheney et al. 2016). However, the mechanisms that cause color and pattern polymorphism in the white *Goniobranchus* with marginal bands species in this study need further examination.

Despite these issues of variability, color and pattern still play important roles in the identification of many nudibranchs, and in at least some *Goniobranchus* species. Based on previous research, putative *Goniobranchus* species that can be identified based on color patterns include *G.splendidus* (Wilson et al. 2016) and the red reticulate species *G.* sp. 1, *G.* sp. 2, *G.* sp. 3, and *G.* sp. 4 from Soong et al. (2020). Additionally, color and pattern-based identification was shown to be useful in all of the species studied here. However, many of the white *Goniobranchus* species with marginal bands are pseudocryptic and have intraspecific color variation which complicates identification, but subtle yet consistent elements of color pattern provide unambiguous features that permit identification of species. This intraspecific color variation was observed in *G.verrieri*, *G.preciosus*, *G.sinensis*, *G.rubrocornutus*, and *G.fabulus* sp. nov. in this study, as well as *G.* sp. 5 in Soong et al. (2020). Thus, based on this study and previous research, at least six molecularly confirmed *Goniobranchus* species have intraspecific color variation, showing that despite confusing color patterns, there are specific morphological characteristics that provide diagnostic features for species identifications. Internal morphological data can help delineate species in *Goniobranchus* and *Chromodoris* (Rudman 1984) and, additionally, molecular data has been able to recover multiple putative species within *Goniobranchus* and other Chromodorididae groups formerly thought to be single species (Matsuda and Gosliner 2018a, 2018b; Layton et al. 2018; Soong et al. 2020; this study). Our study further supports the importance of integrative systematics that both color patterns and internal morphological data is needed with molecular data to aid in nudibranch identification and taxonomy.

Based on the phylogenetic tree in this study (Fig. 1), *Goniobranchusalbonares* was recovered within another clade different from the rest of the white *Goniobranchus* with marginal bands. Most of the white *Goniobranchus* with marginal bands species in this study possibly inherited their white body color with variously colored marginal bands from a common ancestor, except for *G.albonares*, which likely evolved its color pattern independently and convergently. *Goniobranchusalbonares* is very widespread, found in the western Indian Ocean to the western Pacific. Throughout its range, members of the other species with variously colored marginal bands (e.g., *G.daphne*, *G.sinensis*, *G.fabulus*, *G.preciosus*, *G.verrieri*) are ubiquitous and sympatric, ensuring that their mimetic pattern will be present together with other similarly appearing species (Rudman 1985). Gosliner (2001) also noted that a species of polyclad flatworm (*Pseudoceros* sp.) mimicked *Chromodorispreciosa* (*Goniobranchusfabulus* sp. nov. of this study) and that the nudibranchs were far less palatable than the flatworms, suggesting this was a case of Müllerian mimicry.

Well-studied chromodorid nudibranch groups continue to reveal the presence of cryptic species through molecular phylogenetic analyses (Layton et al. 2018). In this study, our examination of *Goniobranchus* species with a white mantle and various marginal bands recovered seven species groups (*G.preciosus*, *G.albonares*, *G.rubrocornutus*, *G.daphne*, *G.verrieri*, *G.fabulus* sp. nov., and *G.sinensis*) of white species with marginal bands. In the past, there has been some confusion regarding the appearance and taxonomy of the *Goniobranchus* species with white mantles and variously colored margins (Rudman 1985; Gosliner et al. 2008, 2015, 2018). Aside from the species and morphotypes examined in this study, there are other described white *Goniobranchus* species with various marginal bands [*G.trimarginatus* (Winckworth, 1946) and *G.galactos* (Rudman & Johnson, 1985)], as well as unidentified morphotypes with white mantles and various marginal bands based on online images (Sea Slug Forum) and field guide books (e.g., Gosliner et al. 2018: 152–154; Nakano 2018: 292–294; Ono and Katou 2020: 195, 196, 200, 202), all of which remain to be examined. There are also white *Goniobranchus* with marginal bands known from Hawaiʻi (Pittman and Fiene 1998), the Marshall Islands (Gosliner et al. 2018: 153, *G.* sp. 26), western Thailand (Gosliner et al. 2018: 153, *G.* sp. 29), New South Wales, Australia (Harasti 2003), the Red Sea (Yonow 1989, 2008), Gulf of Oman (Mayes 2007), the Indian Ocean (Bidgrain 2006), and the South Pacific Ocean (Stenhouse 2000; Potter 2001, 2005). Together, these records suggest a much wider distribution and diversity for this group, and thus further examination is urgently needed to fill in biogeographical gaps and the phylogenetic tree. Therefore, examination of all other described *Goniobranchus* species with these color patterns as well as of other morphotypes, are needed to better understand the relationships between species, and to infer their evolutionary relationships more clearly and better establish robust intraspecific variability thresholds.

## Supplementary Material

XML Treatment for Goniobranchus

XML Treatment for Goniobranchus albonares

XML Treatment for Goniobranchus preciosus

XML Treatment for Goniobranchus rubrocornutus

XML Treatment for Goniobranchus sinensis

XML Treatment for Goniobranchus verrieri

XML Treatment for Goniobranchus fabulus
